# The Great Barrier Reef, a center for Pelagophyceae (Heterokontophyta) diversity, including a new genus and seven new species

**DOI:** 10.1111/jpy.70030

**Published:** 2025-05-28

**Authors:** Richard Wetherbee, Allison van de Meene, Riyad Hossen, Robert A. Andersen, Heroen Verbruggen

**Affiliations:** ^1^ School of BioSciences University of Melbourne Melbourne Victoria Australia; ^2^ Friday Harbor Laboratories University of Washington Seattle Washington USA; ^3^ CIBIO, Centro de Investigação Em Biodiversidade e Recursos Genéticos, InBIO Laboratório Associado, Campus de Vairão Universidade do Porto Vairão Portugal

**Keywords:** Great Barrier Reef, molecular phylogeny, Pelagophyceae, *Revolvomonas*, sand‐dwelling, Sarcinochrysidales

## Abstract

The pelagophytes are a morphologically diverse class of marine heterokont algae defined by deoxyribonucleic acid (DNA) gene sequences, the presence of a multilayered, perforated theca (PT), and the novel role of the Golgi apparatus in the formation and secretion of the PT, as well as materials for the synthesis of the outer extracellular layers (e.g., cell walls and mucilage). We established clonal cultures of sand‐dwelling pelagophytes collected from intertidal and subtidal locations at Heron Island on the Great Barrier Reef (GBR), Australia, and established phylogenetic trees based on nuclear 18S rDNA and plastid *rbc*L, *psa*A, *psa*B, *psb*A, and *psb*C gene sequences that led to the discovery of seven new species and several interesting range extensions. The new genus and species, *Revolvomonas australis*, is sister to *Pituiglomerulus* and *Chrysocystis* in the Chrysocystaceae (Sarcinochrysidales, Pelagophyceae). Additional new species are *Sarcinochrysis kraftii*, *Sa. guiryi*, *Arachnochrysis pilardiaziae*, *A. cassiotisii*, *Sungminbooa capricornica*, and *Su. tropica*; also identified and cultured from the GBR were *Sa. marina*, *Aureoumbra geitleri*, *Chrysoreinhardia giraudii*, *Chrysocystis fragilisi*, and the planktonic *Pelagomonas calceolata*. *Revolvomonas* was studied in detail and has several unusual features for sand‐dwelling pelagophytes. In just three short collecting trips to Heron Island, we were able to isolate and identify over 40% of the pelagophyte genera discovered to date. This study substantiates the diverse nature of pelagophytes and suggests tropical reef sand may be a center for pelagophyte diversity.

**Abbreviations**
GBR: Great Barrier Reef
HPF: high pressure freezing
ML: maximum likelihood
PM: plasma membrane
PT: perforated theca
PV: peripheral vesicles
TEM: transmission electron microscopy

## INTRODUCTION

The class Pelagophyceae was formed when DNA gene sequences revealed a highly unusual, uniflagellate not related to other heterokont classes, with *Pelagomonas calceolata* becoming the type species of the new class (Andersen et al., 1993). Additional studies using molecular markers confirmed that in addition to the order Pelagomonadales, the order Sarcinochrysidales (Gayral & Billard, 1977) belonged to the Pelagophyceae (Han et al., 2018; Saunders et al., 1997). In the space of just 3 decades, the Pelagophyceae has become recognized as one of the most morphologically diverse of all the heterokont classes and is unusual in that all described species are exclusively marine with no freshwater representatives. Furthermore, most species have been observed in benthic habitats along coastlines, with *Pelagomonas* and *Pelagococcus* being the only taxa seen in the open ocean. Currently, the Pelagophyceae contains 22 known genera, with all described species classified within the class based on DNA gene sequences. Additional taxa may well be included in the pelagophytes once several suspect species have been isolated and DNA sequences obtained (e.g., see Han et al., 2018). The history of the phylogenetic position of the Pelagophyceae has recently been described in detail (Daugbjerg et al., 2024; Han et al., 2018; Wetherbee et al., 2022). To summarize, most recent molecular studies consistently placed the Pelagophyceae sister to the Dictyochophyceae, with the diatoms and bolidophytes sister to the Pelagophyceae/Dictyochophyceae clade (Derelle et al., 2016; Dorrell et al., 2017; Dorrell & Bowler, 2016; Han et al., 2018; Wetherbee, Jackson, et al., 2019; Yang et al., 2012).

Pelagophyte species range in size from a 1.5 × 2–3 μm flagellate (*Pelagomonas*) to macroscopic sheets 1 cm across (*Aureoscheda*) and flowing colonies 3–5 cm long (*Chrysocystis*; Andersen et al., 1993; Lobban et al., 1995; Wynne et al., 2014). The planktonic forms are flagellate (*Pelagomonas*, *Chromopallida*, *Plocamiomonas*, and *Veerella*; Andersen et al., 1993; Andersen & Wetherbee, 2024; Daugbjerg et al., 2024; Honda & Inouye, 1995; Wetherbee et al., 2022) or coccoid (*Aureococcus*, *Aureoumbra*, *Pelagococcus*; DeYoe et al., 1997; Lewin et al., 1977; Sieburth et al., 1988), whereas the attached species are predominantly capsoid organisms with vegetative cell division (*Arachnochrysis*, *Chrysocystis*, *Chrysophaeum*, *Chrysoreinhardia*, *Glomerochrysis*, *Pelagospilus*, *Sarcinochrysis*, *Sargassococcus*, *Sungminbooa*, *Wyeophycus*, *Pituiglomerulus*, and an Australian species of *Aureoumbra*; Davison & Bewley, 2019; Han et al., 2018; Wetherbee et al., 2021, 2022). One genus has both a benthic unicellular species and a large colonial species (*Gazia*; Wetherbee et al., 2021), and one genus with a filamentous form is known (*Andersenia*; Wetherbee et al., 2015). One genus has been described from the Arctic region (*Plocamiomonas*), increasing further the range of habitats where pelagophytes have been observed (Daugbjerg et al., 2024).

Recently, pelagophyte genera fixed by high pressure freezing (HPF) and studied with transmission electron microscopy (TEM) were observed to possess a single morphological feature to unite the class, a novel cell covering termed a perforated theca (PT; Wetherbee et al., 2021, 2022). The PT is a multilayered, dense outer covering or sheath penetrated by distinctive pores that encloses benthic cells as well as flagellates and zoospores except where flagella emerge. At least two layers define the PT: a largely transparent, fibrous layer sandwiched between the plasma membrane (PM) and an outer electron dense, perforated layer. Multilayered PTs (four or five layers) were also observed in species eventually assigned to the Pelagophyceae by gene sequencing, and although pores were not mentioned, they were observed in published micrographs (for details, see discussion in Wetherbee et al., 2021). The PT is fabricated by a unique mechanism involving rafts of the PT being synthesized within single Golgi vesicles and injected into established PTs on the cell surface (Wetherbee et al., 2022). In addition, materials destined for the synthesis of the extracellular matrix (cell walls and/or mucilage layers exterior to the PT) were derived by Golgi of different morphology. Golgi‐derived vesicles approached the PM but were never seen to fuse with the PM as is the standard mechanism of deposition for eukaryotic cells. Rather, the PM responded to the presence of vesicles by synthesizing a new section of PM to surround the vesicle. In this manner, vesicles were secreted extracellularly in their entirety where they disintegrated and released their contents between the PM and PT (Wetherbee et al., 2022). Not only is the PT a morphological feature to define the class, but it appears that the function of the Golgi apparatus in extracellular secretion is also novel and has not been observed in any other group of algae that we are aware of.

During our exploration of sand‐dwelling microalgae collected around Australia (Graf et al., 2020; Grant et al., 2011, 2013; Wetherbee et al., 2015, 2021, 2022, 2024; Wetherbee, Jackson, et al., 2019; Wetherbee, Marcelino, et al., 2019; Wetherbee & Verbruggen, 2016), we discovered several unknown pelagophytes. The goal of this study was to describe the pelagophyte flora from tropical Heron Island on the Great Barrier Reef (GBR). We have described seven new species and inferred their phylogenetic position among the Pelagophyceae. We have reported on additional genera observed on Heron Island for the first time, with over 40% of the known genera of pelagophytes having representatives of both major lineages. Our approach consisted of light microscopic observations of culture strains and phylogenetic inference based on multimarker data sets. We also used TEM to observe the PT of a new genus.

## MATERIALS AND METHODS

### Sampling, isolation, and culture

All of the species described in this paper were isolated and cultured from sand samples obtained at various localities around Heron Island, GBR, Australia, on three occasions: September 2022, October 2022, and April 2023. Each sample consisted of ~0.5–1.0 cm^3^ of sand plus seawater that was placed into a 60‐mL culture flask and returned to the laboratory. Clonal cultures were established by isolating benthic cells or zoospores from the field sample by micro‐pipetting into K^−^ enriched seawater medium (Keller et al., 1987). The cultures were maintained in 60‐mL plastic containers at 26°C under Sylvania 58 Luxline Plus and Gro‐Lux fluorescent lamps with a daily 12:12 h light:dark cycle; stock cultures were transferred into new K^−^ medium once a month. To study and film cell divisions and to attempt to induce zoospore production, we introduced fresh medium into a culture in log‐phase 2 h before the end of a dark cycle (12:12 light:dark cycle). Cell divisions, and occasionally zoospore release, occurred 1–2 h after the lights came on in the next light cycle or 15 h later. Alternatively, the process was delayed 24 h at the start of the next light cycle.

### Molecular phylogenetics

To include our samples in the six‐gene phylogenetic framework of pelagophytes introduced by Yang et al. (2012) and Han et al. (2018) and later expanded by Wetherbee et al. (2021, 2022), we carried out short‐read shotgun sequencing. DNA was extracted as described in Cremen et al. (2016). Illumina libraries were prepared with the VAHTS Universal DNA Library preparation kit and sequenced on an Illumina NovaSeq system, paired‐end, with 150 bp reads.

Illumina reads were assembled with the Metaphor workflow (Salazar et al., 2022), with steps for read trimming (fastp, Chen et al., 2018) and sequence assembly (megahit, Li et al., 2015). The contigs containing the six target genes (nuclear 18S rRNA and plastid *psa*A, *psa*B, *psb*A, *psb*C, and *rbc*L) were obtained with BLASTn searches (Camacho et al., 2009), and the genes in question were submitted to Genbank (PQ791806‐63 and PQ788595‐606) and added to the alignments from Wetherbee et al. (2022) using MAFFT 7.520 (Katoh & Standley, 2013) with default settings followed by manual adjustment to retain intact codons.

The six‐gene alignments were concatenated with phykit (Steenwyk et al., 2021), and maximum likelihood (ML) phylogenies were inferred with IQtree 2.3.5 (Minh et al., 2020) with the GTR + F + I + R4 determined as suitable for our data by the built‐in ModelFinder procedure (Kalyaanamoorthy et al., 2017). Branch support was obtained with 1000 ultrafast bootstrap replicates (Hoang et al., 2018). Trees of individual genes were also inferred with IQtree, using the best‐fit model based on ModelFinder and 1000 ultrafast bootstrap replicates.

### Light microscopy

To observe the species, a drop of cell culture was taken at the beginning of the light period, when benthic cells were more likely to be dividing and/or producing zoospores, mounted onto microscope slides with coverslips sealed with a 1:1:1 ratio of Vaseline, lanoline, and paraffin wax. In addition, 5 mL of culture medium containing benthic cells was poured over coverslips in small petri dishes and allowed to settle and grow for 3–14 days. Coverslips were then removed and inverted onto microscope slides and mounted as above. Benthic cells and zoospores (we only observed zoospores in the new genus, not the other new species, although we tried everything we could think of to get them) were observed and recorded using a Zeiss AxioPlan 2 microscope (Carl Zeiss, Oberkochen, Germany), and photographs were taken using a Canon EOS 60D digital single‐lens reflex camera (Canon USA, Melville, New York, United States). Motile cells and zoospores were recorded on video (25 frames per second) and frame grabbed for stills when single photographs were hard to get (e.g., of flagella position and movement).

### Transmission electron microscopy

Cells were prepared by HPF (Wetherbee, Jackson, et al., 2019). Benthic cells were gently centrifuged and placed directly in type A carriers without cryoprotectants and covered with the flat surface of the type B carrier before HPF using a HPF Compact 03 (Engineering Office M. Wohlwend GmbH Switzerland). The cells were freeze substituted in 2% osmium tetroxide and 8% 2,2‐dimethoxypropane (DMP) in acetone for 72 h at −85°C before being slowly warmed to room temperature over the next 2 days. The samples were then washed three times in acetone followed by gradual infiltration in Spurr's resin (Spurr, 1969). Thin (70 nm) sections were cut from pellets of the benthic cell preparations using a Leica UC7 ultramicrotome (Leica Microsystems) and post‐stained with 1% aqueous uranyl acetate and triple lead stain (Hanaichi et al., 1986). The sections were imaged at a voltage of 120 kV using a Tecnai Spirit transmission electron microscope (ThermoFisher Scientific) equipped with an Eagle 2K CCD camera.

## RESULTS

### Molecular phylogeny

**FIGURE 1:**
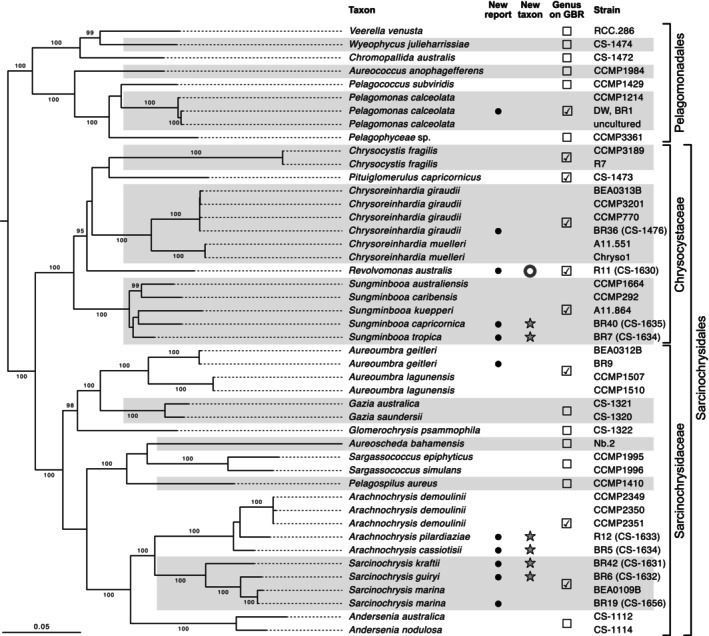
Maximum likelihood phylogeny of the Pelagophyceae indicating the genera we observed on the Great Barrier Reef (tickmarks on the right) and the new taxa described in this paper (stars and donut). The tree was inferred from concatenated 18S rRNA, *psa*A, *psa*B, *psb*A, *psb*C, and *rbc*L gene data. Support values given on the branches are ultrafast bootstraps (listed if ≥95), and the scale bar is in estimated substitutions per site. Outgroups were pruned from the tree for compact visualization. The genus *Plocamiomonas*, for which only 18S rRNA gene data are currently available, was not included.

The ML phylogeny based on concatenated 18S rRNA and plastid *psa*A, *psa*B, *psb*A, *psb*C, and *rbc*L genes showed overall relationships as presented in previous papers, including the major lineages Pelagomonadales and Sarcinochrysidales, with the latter containing the Chrysocystaceae and Sarcinochrysidaceae families (Figure 1). Our strains support the presence of nine out of the 22 currently described genera in the class on Heron Island (GBR; Figure 1). Several of our strains were separated from all known species, supporting the description of six new species (indicated with stars) in existing genera and a new genus containing one additional new species (indicated with a donut). Gene trees of each of the six loci are given in Figures S1–S6. They were mostly in line with the concatenated phylogeny but generally showed lower support and, in some cases, non‐monophyly of one or more of the orders. In the 18S rRNA gene phylogeny, for instance, the Pelagomonadales, along with the genus *Plocamiomonas* (order Plocamiomonadales), formed a basal grade along a poorly resolved ingroup backbone.

### Species descriptions

#### *Revolvomonas australis* Wetherbee gen. et sp. nov. (Figures 2, 3, 4, 5, 6)

*Description*: Marine, colonial thallus alternating with a short‐lived heterokont zoospore stage; colonies spherical with two, four, eight, and up to 64 cells or more in culture, surrounded by a thick wall; the cross‐sectional outline of cells is round to oval (10–12 μm diam.); one or two deeply lobed chloroplasts with pyrenoids; colonies eventually divided one last time, producing zoospores that dispersed as the colony wall broke down, settled, and grew into new colonies. Zoospores oblong (12–16 μm long, 6–10 μm wide); the apical end shaped like a hood or beak with a flat ventral surface and curved dorsal region; the posterior end was rounded; two heterokont flagella emerged from a subapical pit on the ventral surface, one‐third of the cell length below the cell apex; the anterior hairy flagellum (#2) was between 2.25 and 2.5 times the length of the cell (ca. 35–45 μm in length), while the posterior mature flagellum (#1) was about the same length as that of the cells (ca. 14–18 μm); a single chloroplast was multilobed with one or more pyrenoids and located in the posterior of the zoospores; nuclear‐encoded 18S rDNA and plastid‐encoded *rbc*L, *psa*A, *psa*B, *psb*A, and *psb*C gene sequences distinctive from those of other pelagophytes (Figure 1), with GenBank accessions of reference sequences PQ791806‐63 and PQ788595‐606.

*Holotype*: MELUA137648a, a metabolically inactive mounted specimen derived from strain CS‐1630 (R11), collected by R. Wetherbee in April 2023

*Type locality*: Intertidal sand, Heron Island (R11), Queensland, Australia (−23.442368, 151.910678)

*Etymology*: *Revolvomonas* refers to the revolving motion of zoospores during motility and settlement; the specific epithet *R. australis* refers to the country where the species was found, Australia.

*Habitat*: Marine, sand‐dwelling

*Culture lodgement*: ANACC code: Queensland strain CS‐1630, CSIRO, Hobart, Tasmania, Australia

#### *Sarcinochrysis kraftii* Wetherbee sp. nov. (Figure 7a–h)

*Description*: Vegetative cells ovoid (10–12 μm long by 6–8 μm wide) to spherical (mature cells 8–10 μm in diameter, daughter cells 6–8 μm in diam.); cells surrounded by mucilage encased by a gel sheath from the mother cell wall; clusters of cells formed sarcina‐type colonies (i.e., cubic packets of cells) that are loosely arranged and surrounded by grandmother and great grandmother sheaths that stain pink with toluidine blue; daughter cells inherit a single chloroplast that divided into two deeply lobed chloroplasts with finger‐like extensions that spread throughout the cytoplasm; pyrenoids were observed; vegetative colonial reproduction by cluster fragmentation; nuclear encoded 18S rDNA and plastid encoded *rbc*L, *psa*A, *psa*B, *psb*A, and *psb*C gene sequences distinctive.

*Holotype*: MELUA137649a, a metabolically inactive mounted specimen derived from strain CS‐1631 (BR42), collected by H. Verbruggen in September 2022.

*Type locality*: Subtidal sand near the boat channel at a depth of ca. 1 m, Heron Island, Queensland, Australia (−23.442725, 151.909211)

*Etymology*: The specific epithet of *Sa. kraftii* is named after Gerald T. Kraft for his major contributions to the study of seaweeds of Heron Island, the GBR, and around Australia.

*Habitat*: Marine, sand‐dwelling

*Culture lodgement*: ANACC code: Queensland strain CS‐1631; CSIRO, Hobart, Tasmania, Australia

#### *Sarcinochrysis guiryi* Wetherbee sp. nov. (Figure 7i–p)

*Description*: Cells solitary or in pairs, some forming colonies in columns; cells surrounded by mucilage and gel sheaths from mother and grandmother cell walls; sheaths and matrix mucilage only obvious when stained with toluidine blue; cell outlines oval (10–14 μm long by 8–10 μm wide) to round (10–14 μm in diameter); daughter cells round (6–8 μm in diameter); one multilobed chloroplast with thin finger‐like extensions; peripheral vesicles common; pyrenoids in one or more lobes; vegetative colonial reproduction by cluster fragmentation; zoospores not observed; nuclear‐encoded 18S rDNA and plastid‐encoded *rbc*L, *psa*A, *psa*B, *psb*A, and *psb*C gene sequences distinctive.

*Holotype*: MELUA137650a, a metabolically inactive mounted specimen derived from strain CS‐1632 (BR6), collected by H. Verbruggen in September 2022

*Type locality*: Subtidal sand on the reef edge, ca. 2 m deep, Heron Island, Queensland, Australia (−23.443539, 151.908005)

*Etymology*: The specific epithet of *Sa. guiryi* is named after Michael D. R. Guiry for major contributions to the field of phycology, his expertise in algal taxonomy and nomenclature, and for his considerable help to the authors on many occasions.

*Habitat*: Marine, sand‐dwelling

*Culture lodgement*: ANACC code: Heron Island, Queensland strain CS‐1632; CSIRO, Hobart, Tasmania, Australia

#### *Arachnochrysis pilardiaziae* Wetherbee sp. nov. (Figure 8a–j)

*Description*: Cells ovoid (8–10 μm long by 4–5 μm wide) to spherical (6–8 μm in diameter); daughter cells 4–6 μm in diameter immediately after cell division; colonies of cells organized in sarcinoid packets, surrounded by a gelatinous mucilage and gel sheaths; gel sheaths easily observed after staining with toluidine blue; loosely clustered into irregular clumps of colonies surrounded by a prominent sheath of unknown origin; two multilobed chloroplasts with finger‐like extensions, pyrenoids in one or more lobes; peripheral vesicles sparse; vegetative reproduction by fragmentation; zoospores not observed; nuclear‐encoded 18S rDNA and plastid‐encoded *rbc*L, *psa*A, *psa*B, *psb*A, and *psb*C gene sequences distinctive.

*Holotype*: MELUA137651a, a metabolically inactive mounted specimen derived from strain CS‐1633 (R12), collected by R. Wetherbee in April 2023

*Type locality*: Shallow pool, at the boat launch area, Heron Island, Queensland, Australia (−23.441936, 151.910920)

*Etymology*: The specific epithet of *A. pilardiaziae* recognizes the outstanding contributions of Pilar Díaz‐Tapia to the study of algae on Heron Island and Australia more broadly.

*Habitat*: Marine, sand‐dwelling

*Culture lodgement*: ANACC code: Heron Island, Queensland strain CS‐1633; CSIRO, Hobart, Tasmania, Australia

#### *Arachnochrysis cassiotisii* Wetherbee sp. nov. (Figure 8k–t)

*Description*: Cells spherical (6–10 μm in diameter) to slightly ovoid (8–10 μm long by 5–7 μm wide); two, four, or eight or more cells forming sarcinoid colonies; cells surrounded by sheaths from the mother cell wall and colonies surrounded by an outer sheath (grandmother cell wall?); colonies tightly clustered into irregular clumps and surrounded by a sheath of unknown origin; two lobed chloroplasts, pyrenoids present; peripheral vesicles are common; vegetative colonial reproduction by cluster fragmentation; zoospores were not observed; nuclear encoded 18S rDNA and plastid encoded *rbc*L, *psa*A, *psa*B, *psb*A, and *psb*C gene sequences are distinctive.

*Holotype*: MELUA137652a, a metabolically inactive mounted specimen derived from strain CS‐1634 (BR5), collected by H. Verbruggen in September 2022

*Type locality*: Subtidal sand, Heron Island, Queensland, Australia (−23.442725, 151.909211)

*Etymology*: The specific epithet of *A. cassiotisii* recognizes Manny Cassiotis for his continued support and effort in identifying new marine habitats around Australia and widely collecting samples, including on Heron Island.

*Habitat*: Marine, sand‐dwelling

*Culture lodgement*: ANACC code: Heron Island, Queensland strain CS‐1634; CSIRO, Hobart, Tasmania, Australia

#### *Sungminbooa capricornica* Wetherbee sp. nov. (Figure 9a–j)

*Description*: Cells range from spherical (3–5 μm in diameter) to bean shaped (4–6 μm long × 3–5 μm wide); two, four, or occasionally eight cells clustered in sarcinoid packets surrounded by gelatinous mucilage; cells with gel sheaths from the mother and grandmother cell walls; colonies had very loose associations best seen after being stained with toluidine blue; single parietal chloroplast; peripheral vesicles present, often clustered; vegetative reproduction by fragmentation; zoospores were not observed; nuclear‐encoded 18S rDNA and plastid‐encoded *rbc*L, *psa*A, *psa*B, *psb*A, and *psb*C gene sequences distinctive.

*Holotype*: MELUA137653a, a metabolically inactive mounted specimen derived from strain CS‐1635 (BR40), collected by J. Hu in October 2023

*Type locality*: Sand from ca. 1 m depth, at the edge of the boat channel, Heron Island, Queensland, Australia (−23.442507, 151.909675)

*Etymology*: The specific epithet of *Su. capricornica* refers to the Capricorn group of reefs where the species was found.

*Habitat*: Marine, sand‐dwelling

*Culture lodgement*: ANACC code: Heron Island, Queensland strain CS‐1635; CSIRO, Hobart, Tasmania, Australia

#### *Sungminbooa tropica* Wetherbee sp. nov. (Figure 9k–s)

*Description*: Ovoid cells (4–7 μm long by 2.5–5 μm wide) organized into small sarcinoid colonies; cells surrounded by mucilage and cell sheaths from mother and grandmother cell walls; colonies clustered into irregular groups best seen when stained with toluidine blue, one or two large, slightly lobed chloroplasts; peripheral vesicles present, often clustered in groups; vegetative reproduction by fragmentation; zoospores were not observed; nuclear‐encoded 18S rDNA and plastid‐encoded *rbc*L, *psa*A, *psa*B, *psb*A, and *psb*C gene sequences distinctive.

*Holotype*: MELUA137654a, a metabolically inactive mounted specimen derived from strain CS‐1636 (BR7), collected by E. Cassiotis in September 2022

*Type locality*: Sand under the Heron Island jetty, Queensland, Australia (−36.208771, 150.1273)

*Etymology*: The specific epithet *tropica* refers to the Australian tropics where it was collected.

*Habitat*: Marine, sand‐dwelling

*Culture lodgement*: ANACC code: New South Wales strain CS‐1636; CSIRO, Hobart, Tasmania, Australia

### Observations for *Revolvomonas australis*

**FIGURE 2:**
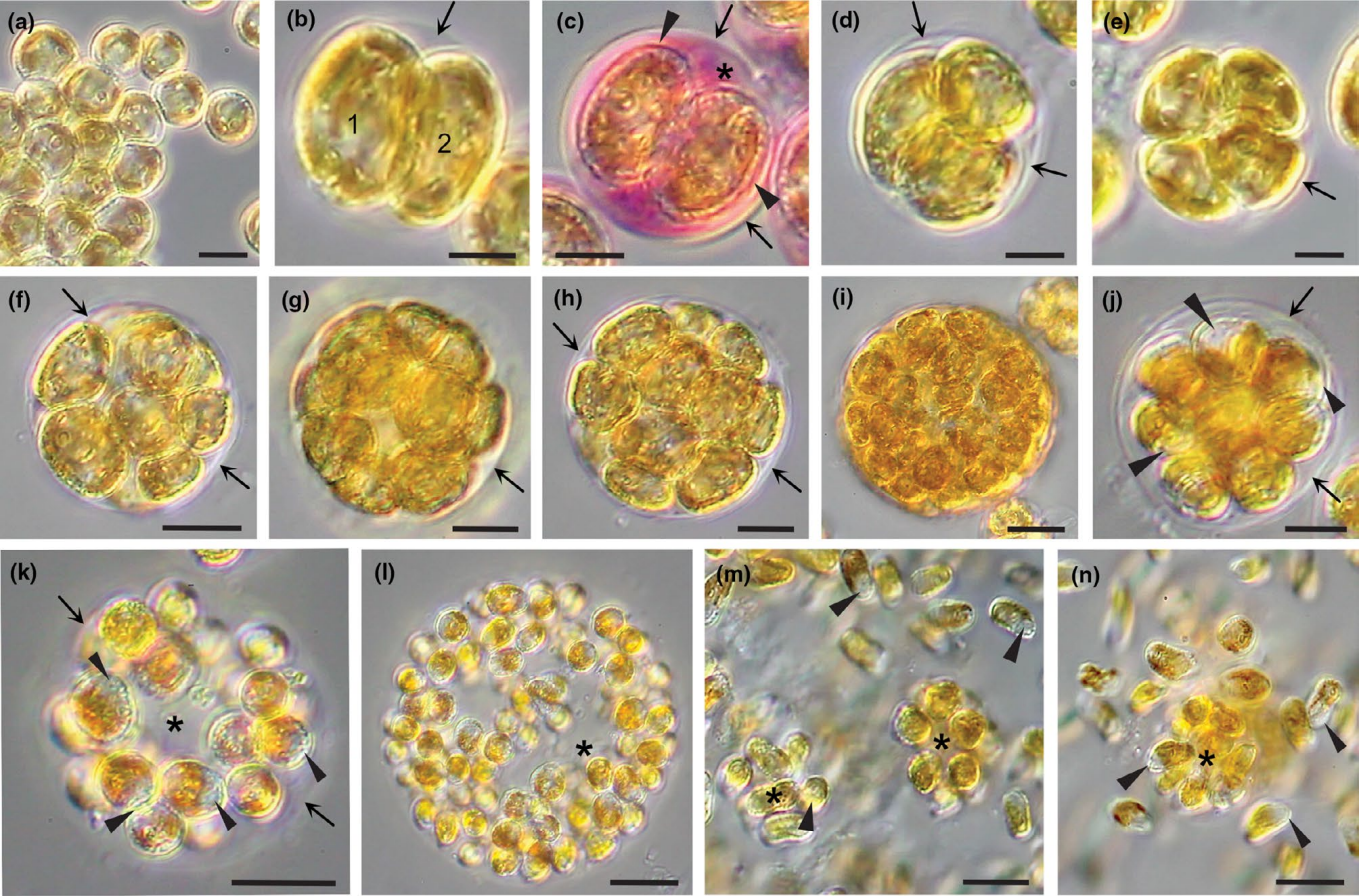
Colony structure and zoospore development in *Revolvomonas australis*. (a) Recently settled zoospores have rounded up and many abut one another, the result of rafting. (b) Two‐celled colony (1 and 2) settled with gel sheath derived from the mother cell wall (arrow). (c) Two‐celled colony stained with toluidine blue showing the PT surrounding each daughter cell (arrowheads), the mother cell wall (arrows), and the mucilage matrix (asterisk). (d–i). Colonies continue to grow (four, eight, 16 cells and more) with the cells tightly packed within the gel casing that is hard to visualize (arrows). (j) Colony cells start to differentiate into zoospores, and they shrink away from the gel casing (arrows) and the future zoospore apical region starts to appear (arrowheads). (k) Overall, colony has close to 32 cells that are all differentiating into zoospores, their colorless apical end is becoming more prominent, and cells are moving toward the outer rim where the casing is no longer seen, although gelatinous matrix is still holding the cells in check. Note the center of the colony is becoming vacant (asterisk). (l) Colony with ~64 differentiated zoospores is held together by a gelatinous matrix. The zoospores have not yet developed flagella, so move through the matrix toward the colony rim. Zoospores are oblong and range between 10 and 16 μm in length and 6 and 10 μm width. (m, n) View at the edge of shrinking mother colonies (asterisk) where many zoospores now have their flagella and are motile, some indicated by arrowheads directed at the cell apex or beak. Cells still isolated in the matrix of shrinking colonies are not yet motile (asterisk). Scale bars: 5 μm (b–e), 10 μm (a, f–j), and 20 μm (k–n).

**FIGURE 3:**
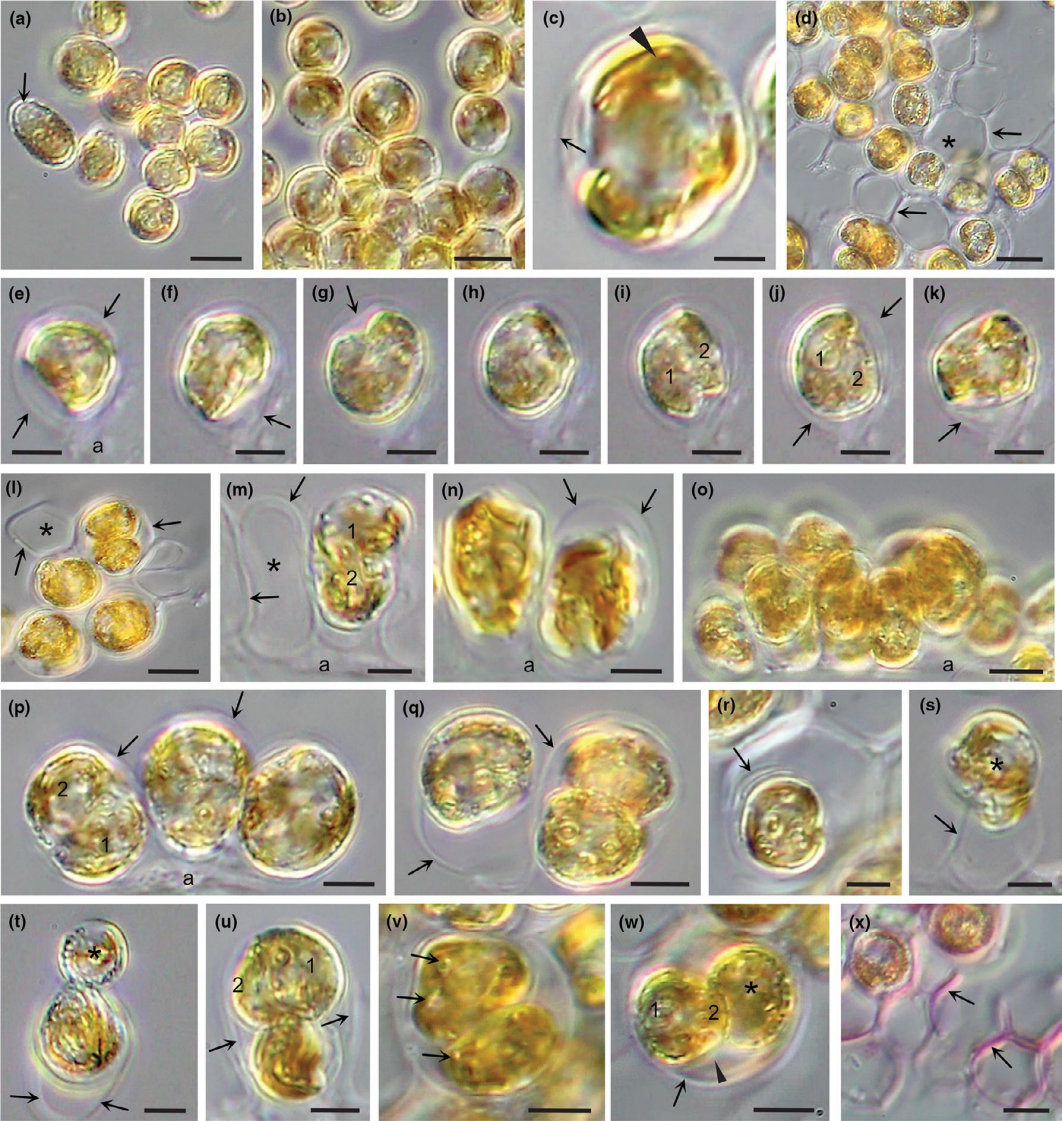
Sporangia structure and development in *Revolvomonas australis*. (a) Recently settled zoospores have rounded and many abut one another, the result of rafting. One zoospore has arrived after the others (arrow). (b) Settled cells have grown, many in rafts. (c) Settled cell with parietal chloroplast with embedded pyrenoids (arrowheads). Cell was rotating, beginning of a thin sporangial wall (arrow). (d) Settled dividing cells with surrounding sporangial walls (arrows) in a honeycomb pattern in cross‐section. Some cells have divided, while other sporangia are empty (asterisk). (e–k). Sequence showing the position of a rotating cell within a forming sporangium, every 30 s for 3 min. Note the adhesive adhering the cell to the slide. Cell appears to be undergoing cytokinesis, the future daughter cells (1 and 2) observed in some images (e.g., i, j), rotating in concert. (l–o) Sporangia and cells in cross‐section and elongated longitudinal view. Note the adhesive (a) binding the sporangia to the substratum. (l) Cross‐section of adjoining sporangia; some empty (asterisk) others with one or two cells. Sporangium walls (arrows) have thickened. (m) One sporangium is now empty (asterisk) with the sporangium wall still intact (arrows); the other sporangium has two cells, while the top cell #1 appears to be leaving. (n) Two sporangia with one cell each. Note, these cells were rotating and the sporangial walls (arrows) were forming. (o) Side view of elongate sporangia that would have a honeycomb structure in cross‐section. (p) Three settled cells next to one another rotating and starting to form a sporangial wall (arrows). One cell appears to be dividing. (q) Two sporangia with walls (arrows) big enough for cells to divide, producing cells that will leave the sporangium and settle elsewhere. (r) A zoospore has settled in an empty sporangium and appears to be secreting its own sporangium wall. (s) A cell appears to be leaving its sporangium, which then would be empty. (t) A cell divides, and one daughter cell (asterisk) appears to be leaving the sporangium. (u) Sporangium with a single cell and a two‐celled colony (1 and 2) about to leave. (v) Glancing section of a two‐celled colony showing pyrenoids in the chloroplast lobes (arrows). (w) Cross‐section of a single cell (asterisk) plus a two celled colony (1 and 2) still in the sporangium. The colony is surrounded by the mother cell wall after division (arrowhead). Note, when the meristematic cell divides, the daughter cells separate completely; although eventually, all cells leave the sporangia and form colonies. (x) Single meristematic cells with their thick sporangial walls plus empty sporangia; the walls all staining pick with toluidine blue and show the honeycomb pattern of this benthic stage. Scale bars: 2 μm (c), 5 μm (a, e–k, m, n, p–x), and 10 μm (b, d, l, o).

**FIGURE 4:**
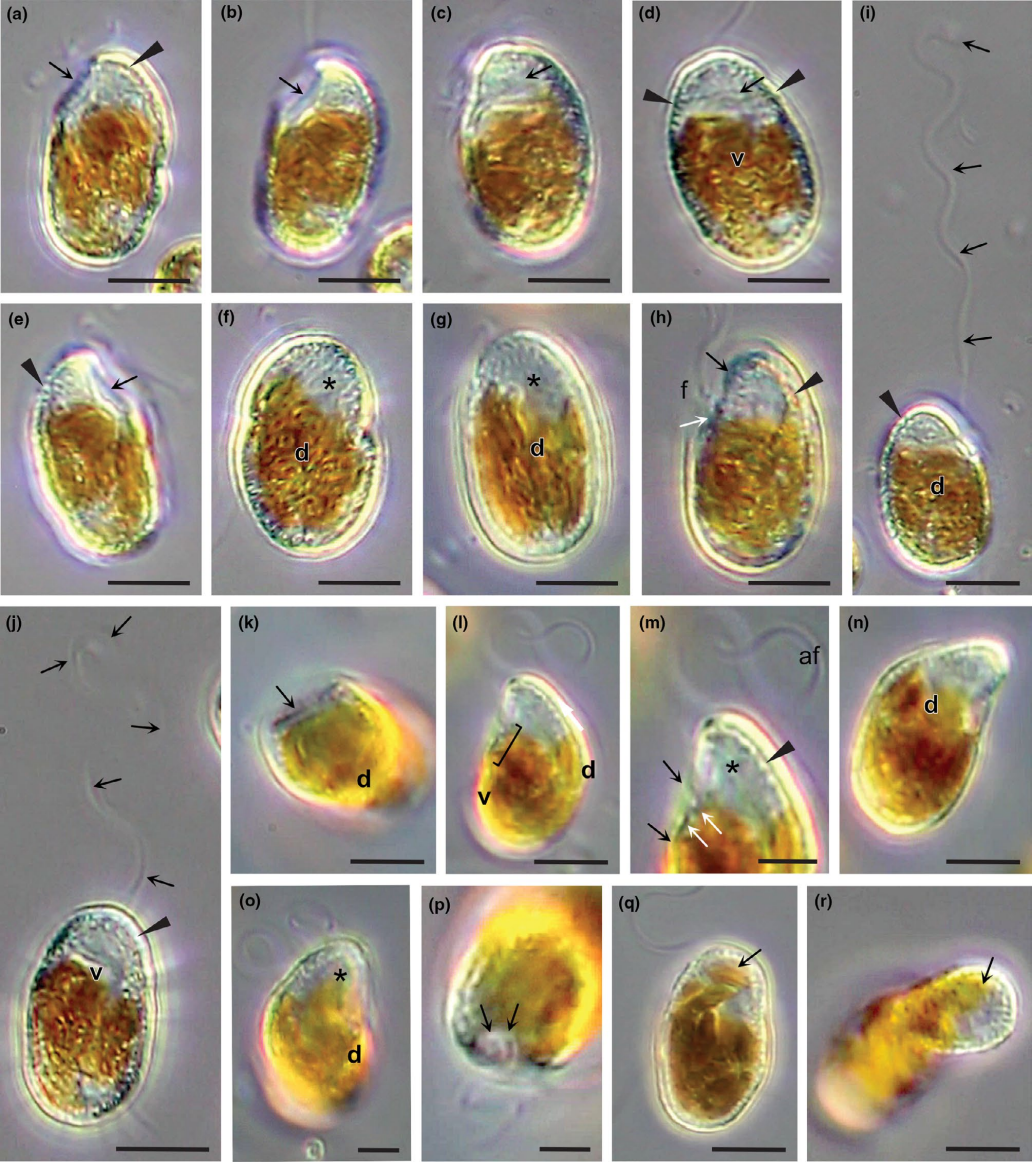
Zoospore structure of *Revolvomonas australis* (a–h) A single zoospore is recorded revolving during motility, in a counterclockwise direction for a full 360°. The apical end of zoospores appears largely transparent in the light microscope and is shaped like a hood or beak, and is flat on the ventral surface (v), as seen from the side (arrows, a, b, e, h, and also k–m), Peripheral vesicles (PVs) are located just beneath the cell membrane, and the flat area is relatively free of PV (arrows, c, d, also j, l, m) but is heavily lined with them (arrowheads, a, d, e, h–j, m), and PVs are common over most of the zoospore, particularly the dorsal side (d) of the beak (e.g., asterisk, f, g) when focused at the cell surface. (i, j) Zoospores filmed moving and rotating slowly through the medium, the long anterior flagellum (arrows) not beating but leading the way. (k) Cross‐section of a zoospore showing the flat ventral surface (arrow). (l, m) Position the flagellar emerge from a pit on the ventral surface just posterior to the flat area of the beak (parenthesis in l) and in close‐up (white arrows, m). The flagella extend in different directions (m, black arrows). Note the PV (arrowhead) on the edge of the flat area of the beak. (n) Dorsal surface (d) of the same zoospore in k–m. (o) In this zoospore, the entire beak region is flattened out (asterisk). Focused in from the apical end of the cell at the position of the flagellar pit where the two flagellar emerge (arrows). (q, r) In some zoospores, a thin lobe of the chloroplast extends up into the cell apex (arrow). Scale bars: 2 μm (m, o, p) and 5 μm (a–l, n, q, r).

**FIGURE 5:**
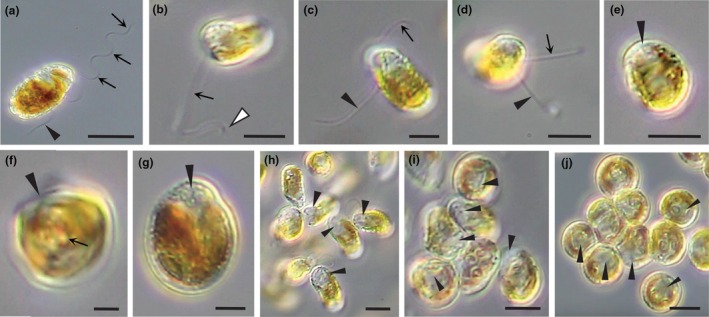
Zoospore settlement of *Revolvomonas australis*. (a) Zoospore showing the anterior flagellum (#2) (arrows) and posterior flagellum (#1) (arrowhead). (b) Zoospore initiates settlement when the anterior flagellum (arrow) adheres to the glass slide (white arrowhead). (c) The posterior flagellum (arrowhead) stops beating, a larger portion of the anterior flagellum (arrow) is adhered to the surface, and the zoospore posterior approaches the surface. (d) The posterior flagellum is in the process of being expelled from the zoospore while some length of the anterior flagellum has been absorbed into the zoospore; the remaining portion adheres to the surface. The anterior end of the cell adheres, and the zoospore flattens out on the surface. (e) Settled zoospore after 5 min. Note the apical end of the cell is still obvious (arrowhead). (f) Prior to settlement zoospores spin on an axis (arrow) at the posterior end of the cell before suddenly stopping settling. Note the flattened ventral surface of the beak (arrowhead). (g) Settled cell in previous figure (f), with the former apical end of the cell observed (arrowhead). (h–j) Zoospores often settle together in rafts, adhering with their posterior ends; the former apical beaks (arrowheads) are observed following settlement (h, i) and an hour later when settlement is complete, the beginning of the benthic stage (j). Scale bars: 2 μm (f, g) and 5 μm (a–e, h–j).

*Light microscope observations*: Thalli consisted of spherical, benthic colonies ranging in size from two to 64 cells or more (Figure 2). The dominant benthic stage alternated with a short‐lived zoospore stage. Zoospores settled, often together in rafts, and formed spherical benthic cells with one or two lobed parietal chloroplasts (Figures 2a and 3a–c). These cells enlarged and became more ovoid in shape and developed in two different ways, both ultimately resulting in new colonies (Figures 2, 3, 4, 5).

In the first method, zoospores settled, adhered, and started cell division cycles, forming multicelled colonies surrounded by a sheath derived from the initial mother cell wall (arrows, Figures 2b–i and 3v,w). Colonies were packed with cells, and the wall was difficult to observe without staining (Figure 2c–i). Colonies were not perfect spheres, as the region abutting the substratum was flattened out, and the adhered cells secreted an adhesive that bound the spheres to a surface. Eventually, by some unknown signal or stimulus, all cells divided one last time and produced zoospores (Figure 2j–n). Colonies whose cells had started to differentiate into zoospores were initially identified by the appearance of a nonpigmented region in cells that later translated into the apical beak of zoospores (Figures 2j,k and 4a–r). As zoospores differentiated, the colonies became swollen, and the cells migrated toward the covering of the colony by amoeboid movement as flagella were not yet functional (Figure 2k,l). The covering wall slowly disintegrated, but the developing zoospores did not have full motility and remained within the outline of the former covering and seemed enmeshed in a matrix of mucilage prior to slowly escaping the colony. Eventually, flagella matured and zoospores became motile (Figure 2m), swimming to a substratum for settlement. It took up to 30 minutes or longer for all the zoospores to escape the mother colony and become motile.

In the second method of generating new benthic colonies, settled zoospores did not transition directly into colony formation, but rather differentiated into a thick‐walled sporangium (Figure 3a–q). Settled cells enlarged and produced an adhesive layer (a, Figure 3e,m–p) that established a position on the substratum, often in rafts created by the settling zoospores. Cells then spontaneously rotated in three dimensions, rapidly changing shape (Figure 3e–k), an action that coincided with the formation of a sporangial wall with a similar diameter as the rotating cell but elongated with room for cell divisions and the accumulation of new cells (Figure 3d–r). Occasionally, cells divided; the daughter cells looked and acted in the same manner (Figure 3i,j). The sporangial walls thickened and, together with adjacent sporangial walls, appeared honeycombed in cross‐section (Figure 3d,l,r) and stained pink with toluidine blue (Figure 3x). Once the sporangial wall was established, the enclosed cell(s) divided to produce a new population of benthic cells that left the sporangium (Figure 3m,s,t) and divided to produce a two‐celled colony that further developed into a colony elsewhere (Figure 3u).

Regardless of whether colonies were derived directly from zoospores or from benthic sporangia, subsequent development into multicellular colonies was the same. The first division resulted in daughter cells that were imbricated and enclosed by a mother cell wall (Figure 3u–w), eventually dividing to produce a four‐celled colony, then an eight‐celled colony, and so forth. All cells eventually left the sporangium and formed new colonies, which resulted in empty sporangial walls (Figure 3d,l,m). Empty sporangial walls could be reinhabited by zoospores from a subsequent zoospore release, settle, and then rotate to produce a new sporangium within the old one (arrow, Figure 3r).

Zoospore morphology was unusual for a pelagophyte, and zoospores utilized two very different modes of motility. Zoospores were characterized by a distinctive apical end that was shaped like a hood or beak, being flat on the ventral surface and rounded on the distal surface (Figure 4a–r). Peripheral vesicles were common over most of the zoospore surface but scarce on the ventral face of the beak (e.g., Figure 4c,d,j,l,m). The beak comprised a quarter to a third of the apical end of zoospores and looked transparent in the light microscope, usually lacking a lobe of the chloroplast. The posterior was rounded and wider than the anterior beak. Overall, zoospores were ca. 12–16 μm long by 6–10 μm wide, while a large, lobed chloroplast dominated the bottom two‐third of cells.

Two heterokont flagella arose from a small pit on the bottom edge of the beak ventral surface (Figure 4h,l,m,p). The anterior flagellum (#2) was 2.25–2.5 times the length of the cells (ca. 35–45 μm, Figures 4i,j and 5a), while the posterior flagellum (#1) was about the length of the cells (ca. 14–18 μm, Figure 5a). Zoospore motility was generated in two different patterns by flagella movement, the first like most heterokonts, with the anterior flagellum generating a sinusoidal wave that resulted in forward movement. Alternatively, zoospores also moved slowly, with the anterior flagellum loosely leading in front of the cells but not beating (Figure 4i,j). As zoospores both revolved and moved only by the action of the posterior flagellum, motility was relatively slow and easily observed and measured. Cells revolved a full 360° ca. 2.5 times a second, often stopping and then revolving in the opposite direction. They also changed to half revolutions, revolving in one direction for 180° and then back again to where they started, or occasionally, zoospores moved forward without revolving at all. The morphology of a single zoospore was observed in one counter‐clockwise revolution (Figure 4a–h) and showed the characteristic beak with a flattened ventral surface and the origin of the flagella. In some zoospores, a lobe of the chloroplast was observed to extend up the dorsal side of the beak (Figure 4q,r).

Zoospores were motile for up to 1 h, and settlement was initiated by the long, anterior flagellum attaching to a coverslip at or near its tip (Figure 5b,c). After attachment, zoospores exhibited a jerking motion, the movement apparently generated by the short posterior flagellum as the anterior flagellum was stiff and gradually being resorbed by the cell, drawing the zoospore closer to the surface (Figure 5b–d) where the two flagella seemed to manipulate the zoospore so that the posterior end was drawn to the substratum. The anterior beak was always free in the medium. In many cases, cells revolved quickly on an axis once they touched the surface at the posterior end of the cell (Figure 5f) at ~2.25–2.5 revolutions per second for 1–2 min, before adhering and flattening out on the surface (Figure 5g). The spinning action was not always observed if the zoospores were crowded or competing for a position in a raft. Once adhered, the zoospores expelled the posterior flagellum into the medium, while a fragment of the adhered anterior flagellum often remained. The zoospores tended to settle together in rafts where they flattened out over the surface forming a spherical to ovoid benthic cell where the beak area was still initially visible (arrowheads, Figure 5h–j). The new benthic cells then followed one of the two methods of differentiation discussed above.

**FIGURE 6:**
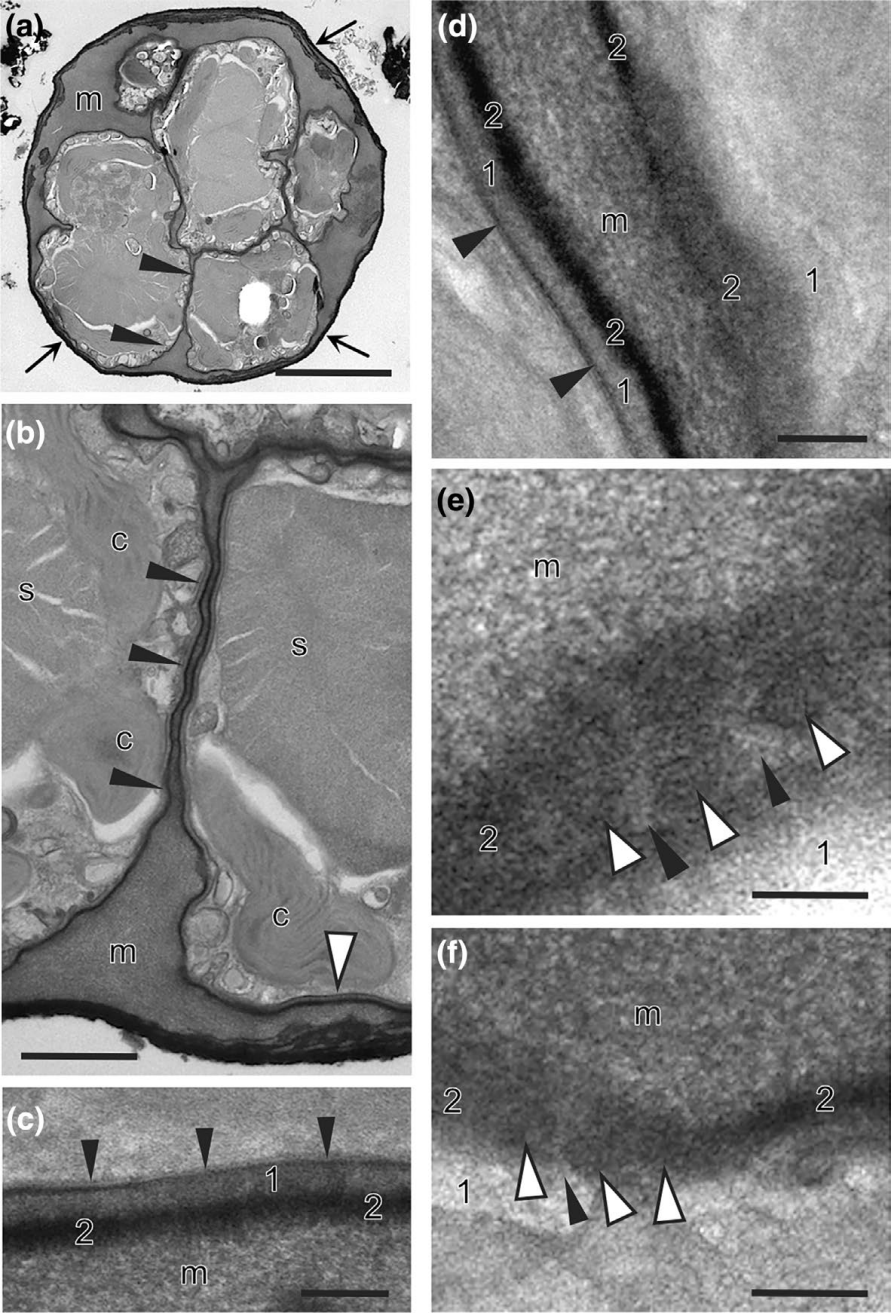
Fine structure of *Revolvomonas australis*. (a) Section through a small colony showing the cells immersed in mucilage (m) and surrounded by a thick covering (arrows). The PT is obvious even at this low magnification as a dense layer defining cell shape (e.g., arrowheads). The area where two cells are adjacent to one another (arrowheads) is shown in (b) at higher magnification. (b) Two adjacent cells where their PTs meet with a thin layer of mucilage separating them (arrowheads). Chloroplasts and large storage products (s) of unknown composition are observed. The white arrow indicates a section of the PT shown in (c) at a higher magnification. (c) Bilayered PT, the PM (arrowheads) and electron dense, porous layer #2 sandwiching layer #1 that is fibrillar and more lightly stained. Layer #2 is packed with micropores about the thickness of the PM. (d–f). The structure of the PT in both cross‐section (d) and glancing section (d–f). Layer #2 is porous and has bunches of micropores (white arrowheads) with lighter stained regions between as seen in (e, f) (black arrowheads). Layer #1 also appears to have pores in glancing section. Scale bars: 100 nm (a) and 5 μm (e–h, m–p).

*Fine structure*: The HPF fixation of *Revolvomonas australis* was excellent for most cell features (Figure 6a,b), particularly the PM and associated PT (Figure 6c–f). As with other genera from the Sarcinochrysidales, *R. australis* had a bilayered PT with both layers of the same thickness (40–50 nm) and following the contours of the cell (Figure 6b–d). Layer #1 was lightly stained and fibrous, with some fibers running perpendicular to the PM and layer #2, which was electron dense and porous, packed with micropores with a diameter of ca. 10–12 nm, often in small groups, as seen in glancing sections (Figure 6e,f).

### Observations for *Sarcinochrysis kraftii* and *Sa. guiryi*

The gene sequences were distinctive between the two new species described here and between each new species and the type species, *Sarcinochrysis marina*.

**FIGURE 7:**
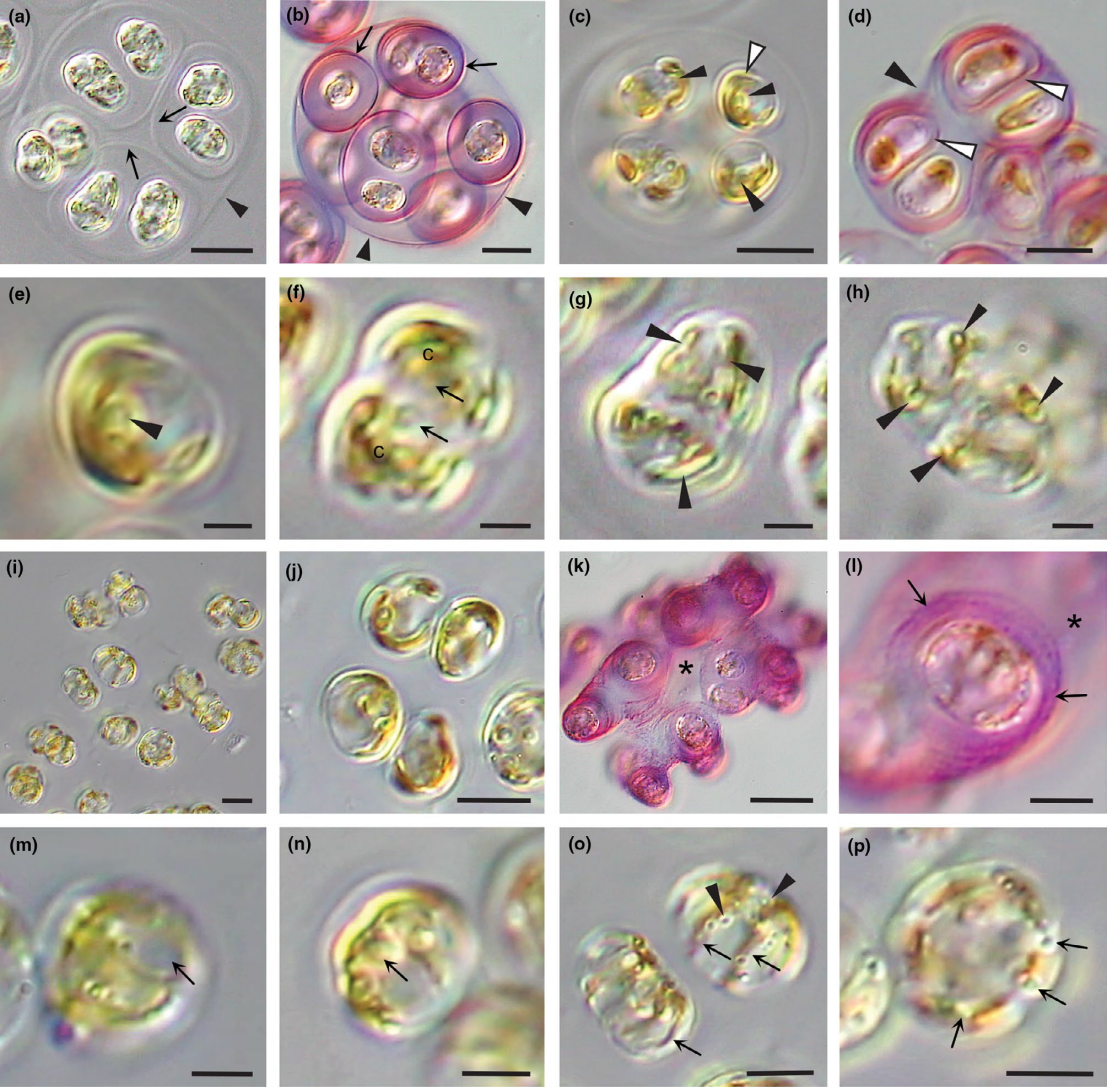
*Sarcinochrysis kraftii* (a–h) and *Sarcinochrysis guiryi* (i–p). (a) Four pairs of ovoid cells surrounded by mucilage and gel sheaths from the mother cell wall (arrows), further enclosed a grandmother gel sheath (arrowheads). (b) Large colony with gel sheaths and mucilage surrounding cells stained pink with toluidine blue. The grandmother gel sheath (arrowheads) and mother cell sheaths (arrows) are more heavily stained. (c) Four small daughter cells following division have a single u‐shaped chloroplast (c) that divides into two, each with a pyrenoid (arrowhead). (d) Cell walls (white arrowheads) and cell sheath (black arrowhead) stained pink with toluidine blue. (e) High magnification image of a cell in (c) (white arrowhead) with a pyrenoid (arrowhead). Note, a second chloroplast is out of focus. (f) Dividing cell with two deeply lobed chloroplasts (c) and two daughter nuclei (arrows). (g and h) Mature cells with arms of the lobed chloroplasts shown in longitudinal and cross‐section (arrowheads). (i and j) *Sarcinochrysis guiryi* cells generally single or are in pairs with one deeply lobed chloroplast. (k and l) Cell pairs surrounded with mucilage and often incomplete gel sheaths (arrows) form colonies composed of columns that stain pink with toluidine blue. Note cells in (l) are dividing. (m) Nucleus located between lobes of the chloroplast. (n) Pyrenoids in lobes of the chloroplasts (arrows) (o and p) Thin finger‐like lobes of the chloroplast (arrows) are seen in both longitudinal section and cross‐section. Peripheral vesicles (arrowheads) are scattered near the cell surface (arrowheads in o). Scale bars: 10 μm (a–d, i–l) and 5 μm (e–h, m–p).

The morphological features of *Sarcinochrysis kraftii* (Figure 7a–h) vegetative cells were very similar to those of *Sa. marina*, although we did not see the zoospores for comparison. Vegetative cells were in sarcinoid packets of four, eight, or more cells (Figure 7a), surrounded by mucilage and gel sheaths from mother cell walls and further by grandmother gel sheaths, best seen in colonies stained with toluidine blue (Figure 7b,d). Note a lightly stained matrix that surrounded the colonies. Cells were ovoid (10–12 × 5–7 μm) to spherical (7–8 μm in diameter) in shape. Following division, cells were ovoid (8–10 × 5–7 μm) to spherical (7–8 μm in diameter; Figure 7c,e). Cells had two deeply lobed chloroplasts (arrows, Figure 7f,g) with pyrenoids (arrowheads, Figure 7c,e). Note the two nuclei (arrows, Figure 7f) in a cell that was dividing. Peripheral vesicles were not observed in this species.

*Sarcinochrysis guiryi* was distinct from *Sa. marina* and *Sa. kraftii*, as cells were not clustered into sarcinoid packets, but one or two cells were dispersed in a sticky mucilage (Figure 7i,j) as seen in light microscopy. Cells were surrounded by mucilage and gel sheaths from old mother cell walls but were only observed after heavy staining with toluidine blue. Columns of cell pairs, seen in cross‐section (Figure 7k), were surrounded by a stained matrix (e.g., asterisk, Figure 7k,l). Cells had a single parietal chloroplast with multiple thin lobes extending out into the cytoplasm (arrows, Figure 6o,p). Pyrenoids were sometimes observed (e.g., Figure 7n). Small numbers of peripheral vesicles were present (arrowheads, Figure 7o). Note the nucleus located between the chloroplast lobes (arrow, Figure 7m).

### Observations for *Arachnochrysis pilardiaziae* and *A. cassiotisii*

**FIGURE 8:**
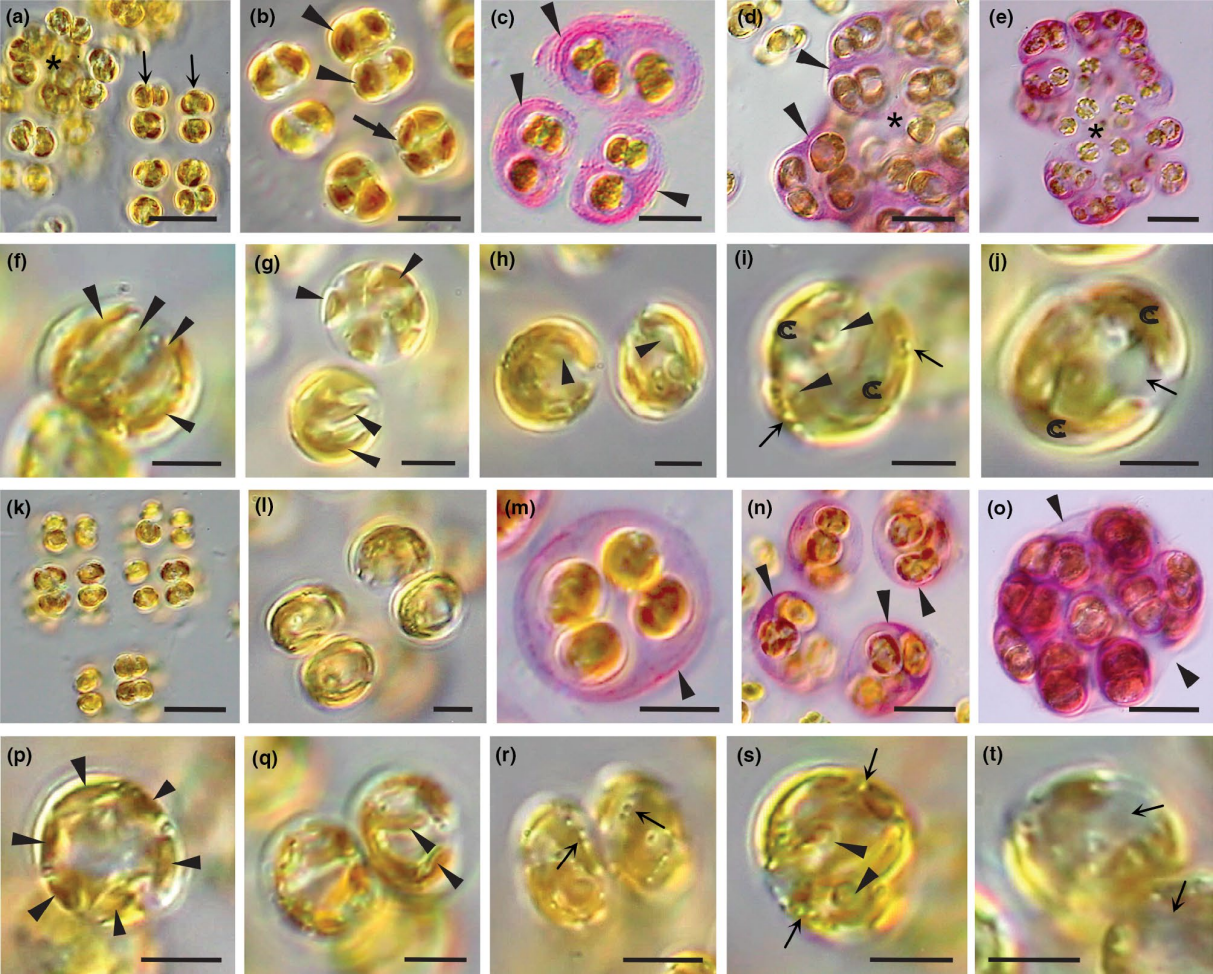
*Arachnochrysis pilardiaziae* (a–j) and *Arachnochrysis cassiotisii* (k–t). (a) Adhering packets of cells, either in rows (arrows) of four cells each or packets with bunches if cells (asterisk). (b) Cells oval to rounded; two chloroplasts (arrowheads) becoming four prior to division (arrow). (c–e) Cells surrounded by mucilage and gel sheaths (arrowheads) seen after staining with toluidine blue. Cell packets, typically of four cells, are held together by a matrix into large, irregular clumps of cells with an outer sheath of unknown origin (arrowheads in d). (f and g) Chloroplasts deeply lobed (arrowheads). (h and i) Pyrenoids observed in chloroplast lobes (arrowheads); peripheral vesicles (arrow) scarce adjacent to the cell membrane. (j) Nucleus (arrow) is observed between lobes of the chloroplast. A. (k and l) Colonies comprised of two, four, or more cells separated by mucilage to form large sarcinoid colonies. (m, n) Colonies stained by toluidine blue reveal cells surrounded by pink mucilage and gel sheaths (arrowheads). (o) Colonies can fuse to form large clumps that are surrounded by sheaths of unknown origin (arrowheads). (p, q) Cells contain two deeply lobed chloroplasts (arrowheads). (r) Peripheral vesicles (arrows) adjacent to the cell membrane are common. (s) Pyrenoids are observed in lobes of the chloroplasts (arrowheads). (t) Nuclei (arrows) of two cells are shown between chloroplast lobes. Scale bars: 20 μm (a, d, e, k, n, o), 10 μm (b, m, p, q, r), and 5 μm (c, f–j, l, s, t).

The gene sequences were distinctive between the two new species as well as between each new species and the type species for this genus. *Arachnochrysis pilardiaziae* consists of classic sarcinoid packets of four, eight, and more cells packed into clusters and surrounded by a sticky mucilage that binds them to a substrate (Figure 8a–e). Gel sheaths derived from old mother and grandmother cell walls were observed when stained with toluidine blue (Figure 7c–e), while large clusters of cell packets were encased in a lightly stained matrix (asterisk, Figue 8d,e) and surrounded by a wall of unknown origin (arrowheads, Figure 7d). Vegetative cells were spherical to slightly ovoid, 7–10 μm in diameter (Figure 8f–j), while recently divided cells were ovoid, 10–12 × 5–6 μm. Two chloroplasts were deeply lobed (Figure 8f–j) with pyrenoids (arrowheads, Figure 8h,i). Peripheral vesicles were sparse (arrows, Figure 7i). Note the nucleus between two chloroplast lobes (arrow, Figure 8j).

Vegetative cells of *Arachnochrysis cassiotisii* were arranged in four, eight, and more sarcinoid packets (Figure 8k–n) and surrounded by mucilage and gel sheaths from old mother and grandmother cell walls, best observed when stained with toluidine blue (arrowheads, Figure 8m,n). Packets adhered to one another to form compact clusters surrounded by a stained matrix and a wall of unknown origin (arrowheads, Figure 7o). Cells were round or slightly ovoid, 8–10 μm (Figure 8p–t) and contained two deeply lobed chloroplasts (arrowheads, Figure 8p,q) with pyrenoids (e.g., arrowheads, Figure 8s). Peripheral vesicles were common (arrows, Figure 7r,s). Note the nuclei located between the chloroplast lobes in two cells (Figure 7t).

### Observations for *Sungminbooa capricornica* and *Su. tropica*

**FIGURE 9:**
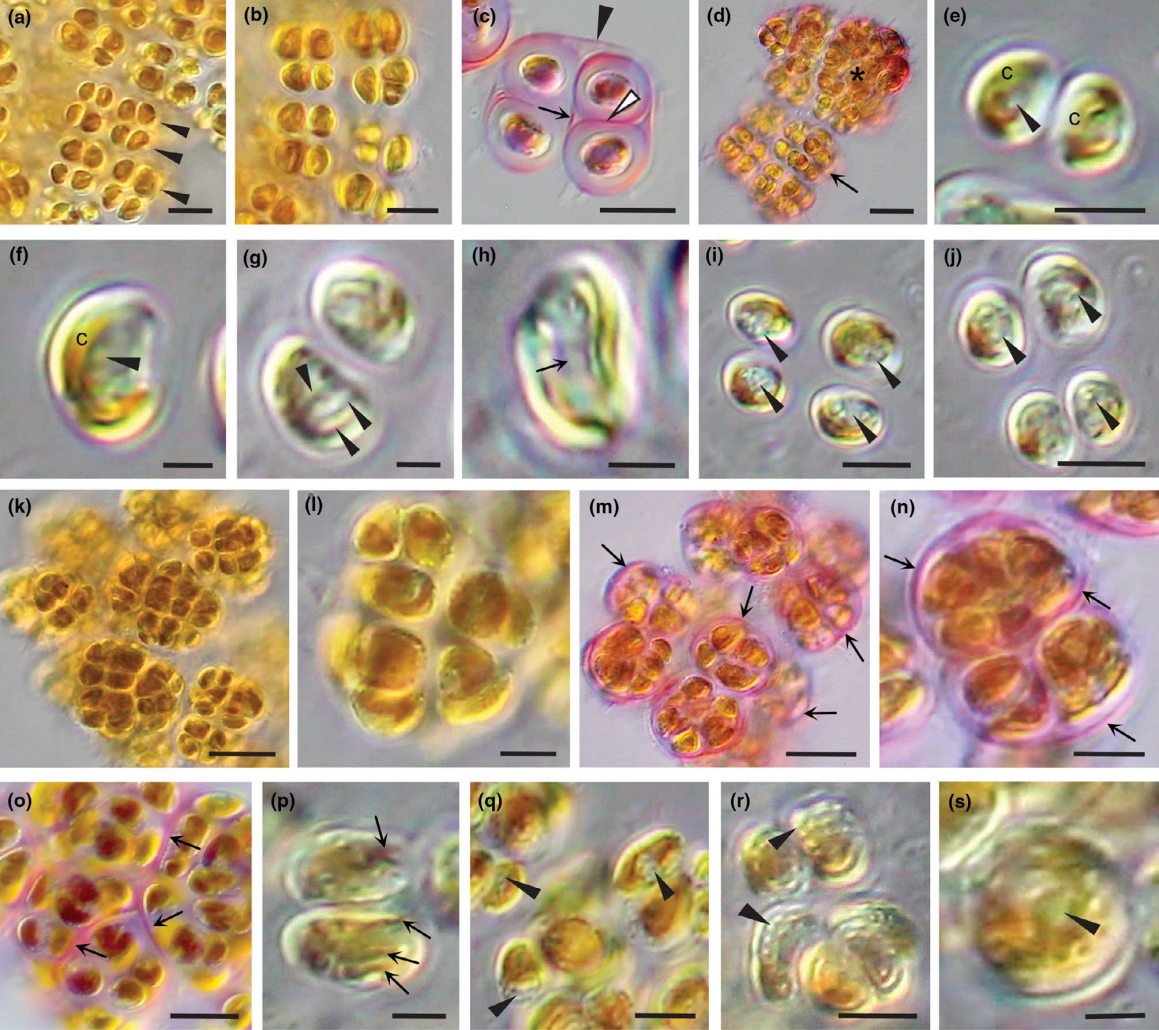
*Sungminbooa capricornica* (a–j) and *Su. tropica* (k–s). (a and b) Packets of four, eight, and more cells aligned in rows (e.g., arrowheads, a) to form sarcinoid colonies. (c and d) Cells stained with toluidine blue. (c) Cells surrounded by mucilage and gel sheaths from mother (white arrow), grandmother (black arrow), and great grandmother (arrowhead) cell walls. (d) Sarcinoid colonies (arrow, d) form irregular clusters when cells continue to divide, thus losing the cubic nature of the packets (asterisks, d). (e and f) Cells have a single chloroplast with a pyrenoid (arrowheads). (g) Chloroplasts are lobed (arrowheads). (h) The nucleus is centrally located (arrow). (i and j) Peripheral vesicles are common, often concentrated in groups away from the chloroplast lobes (arrowheads). S. #2 (k and l) Sarcinoid colonies are large and composed of colonies (16, 32, or more cells). (m–o) Colonies stained with toluidine blue and surrounded by gel sheaths and bunched together with a lightly stained matrix holding the colonies together. Note in (o), compact clumps of colonies with thickened cell sheaths within (arrows). (p) Chloroplasts lobed (arrows). (q and r) Peripheral vesicles common, often in groups between chloroplast lobes (arrowheads). (s) Nucleus in the center of the cell (arrowhead). Scale bars: 20 μm (a, d, k, m, o), 10 μm (b, c), 5 μm (e, i, j, i, n, q, r), and 2 μm (f–h, p, s).

The gene sequences were distinctive between the two new species described here and among the three other described species (Figure 1). *Sungminbooa capricornica* had cells in two‐, four‐, and eight‐celled packets in sarcinoid packets (Figure 9a,b), surrounded by mucilage and gel sheaths from old mother and grandmother cell walls that were best seen after staining (Figure 9c,d). Cells continued to divide, creating cell masses rather than packets (asterisk, Figure 9d). Packets were loosely associated, but a matrix did not stain (Figure 8d). Cells were bean shaped, 4–6 × 3–4 μm (Figure 9a–j), to spherical and contained a single‐lobed chloroplast (c) with a pyrenoid (arrowhead, Figure 9e–g). The nucleus was centrally located (arrow, Figure 9h), and peripheral vesicles were common, often bunched together in regions where the chloroplast was absent (e.g., arrowheads, Figure 8i,j).

*Sungminbooa tropica* consisted of four, eight, 16, and more cells formed into sarcinoid packets that tightly packed together to form large clusters (Figure 9k–o), surrounded by a thick covering of unknown origin (Figure 9n). Cells in packets continued to divide, producing clumps of cells rather than the cubic packets first seen (Figure 8m–o). Cells were oblong (8–10 × 4–5 μm) to spherical (4–6 μm) and surrounded by mucilage and gel sheaths (Figure 9m–o). Cells contained a large parietal chloroplast with a few short lobes or extensions (arrows, Figure 9p). Pyrenoids appeared to be present, while peripheral vesicles were common, often concentrated in groups (arrowheads, Figure 9q–s). Note the nucleus (arrowhead, Figure 9s).

### Range extensions

*Sarcinochrysis marina*, *Aureoumbra geitleri*, *Chrysoreinhardia giraudii*, *Chrysocystis fragilis*, and the planktonic *Pelagomonas calceolata* were isolated from Heron Island samples, and gene sequences were used to establish their identity. We reported on *Chrysoreinhardia giraudii* from Heron Island in a previous paper (Wetherbee et al., 2022), and in this paper, we report on the isolation of the other four species for the first time and comment on their overall distribution worldwide, including Australia.

## DISCUSSION

### Molecular phylogenetics

The strong statistical support obtained in our six‐gene molecular phylogeny delivered a solid framework to interpret relationships among pelagophyte species. It is important to note that one genus, *Plocamiomonas*, was not included in our main phylogeny, as only the 18S rRNA gene is publicly available for the genus, with none of the five plastid markers available for inference of multigene phylogenies. Based on 18S rRNA gene topologies, this genus appears as an early branching lineage within the class, sister to a clade containing Pelagomonadales and Sarcinochrysidales, and a separate order (Plocamiomonadales) was defined to contain it (Daugbjerg et al., 2024).

The taxonomic decisions made in our paper are strongly supported by the six‐gene phylogeny. The new genus *Revolvomonas*, which was nested in the Chrysocystaceae, was sister to a clade containing multiple genera (*Chrysoreinhardia*, *Chrysocystis*, and *Pituiglomerulus*), forming clear evidence of its distinctive nature at the molecular level, with the long branch on which it resides indicating a level of divergence from other genera on par with that among other genera in the Pelagophyceae. The two new species of *Sungminbooa* (*Su. capricornia* and *Su. tropica*) were distinct from the other species in that genus, with levels of divergence similar to those among other species in the genus. It is worth noting that the relationships among *Sungminbooa* species were not well resolved in our six‐gene phylogeny, with only the *Sungminbooa australiensis–Sungminbooa caribensis* sister relationships receiving strong support, but the relationships among remaining species remain in doubt. Within *Arachnochrysis*, the two proposed species (*A. pilardiaziae* and *A. cassiotisii*) are clearly separated from *A. demoulinii*, the only other species described in the genus, with levels of divergence on par with those observed in other genera of pelagophytes. Similarly, the two proposed new species in *Sarcinochrysis* (*Sa. kraftii* and *Sa. guiryi*) are distinctive from *Sa. marina*, the only species described in that genus, with levels of divergence among the species comparable to those in *Arachnochrysis*.

### Morphology and development of *Revolvomonas*

The first sign of zoospore differentiation came when the distinctive beak region appeared in the still rounded cells that pulled away from the colony covering (i.e., Figure 2j). This differentiation began toward the end of the dark period, ~2–3 h prior to the start of the release of zoospores. Colonies then swelled in size, the covering started to disintegrate, and zoospore development continued as described in the Results section. It took considerable time for all the zoospores of a large colony to successfully mature and migrate out of the colony matrix and become motile. Increasing the dispersal time of a single colony in a challenging tide pool habitat, where environmental conditions can change instantly, would increase the chances of settlement by at least some of the colony's zoospores.

*Gazia saundersii* is another pelagophyte species that has a colonial benthic stage that looks somewhat like *Revolvomonas australis* but has two mechanisms for dispersing its contents. First, cells within the colonies of *G. saundersii* are organized into smaller baby colonies surrounded by their own wall. Most of the baby colonies from one mother colony contain the same number of cells and can be released as such when the mother colony wall breaks down (Wetherbee et al., 2021). The second mechanism involves the large colonies (120 plus cells) of *G. saundersii* producing motile heterokont zoospores that were released together in a mass exodus, not like the steady stream of escaping zoospores seen in *R. australis (*Wetherbee et al., 2021). The two methods (i.e., by baby colonies or zoospores) likely increase dispersal and survival, but how *G. saundersii* colonies know when to develop one way or the other is not known. What we do know is that all colonies of *G. saundersii* in a single culture react at the same time and in the same way, differentiating and releasing either baby colonies or zoospores. Similarly, *R. australis* cultures had a synchronized formation and release of zoospores. As the light and temperature conditions were constant in our culture chambers, colony differentiation presumably has something to do with nutrient depletion, colony density, accumulation of triggering biomolecules, and so forth. It is hard to imagine that is the case in tide pool sand where colonies would be replenished by the tides twice a day, thus suggesting that cultures respond differently than natural populations or that several interrelated physical conditions probably come into play.

### Zoospore movement and dispersal in *Revolvomonas*

Interestingly, zoospores displayed two different modes of motility that we had not observed in other pelagophytes. Classic heterokont motility occurred after release from the mother colony, the anterior flagellum (#2) pulling the zoospore behind at a speed that makes observations limited. The second mode of motility was much slower and methodical and allowed us to observe the zoospores in some detail. The shorter posterior flagellum (#1) generated both the forward movement and zoospore revolutions, while the anterior flagellum (#2) did not beat, projecting out in front of the cell, revolving at the same rate as the zoospore while occasionally touching the surface at its tip. The zoospores underwent between 2.25 and 2.5 revolutions per second, alternating the direction of the revolutions between clockwise and counterclockwise, or then rocking back and forth 180°, or not revolving at all. The two modes of motility appeared to work in concert with one another; the classic heterokont motility generates high speed for the zoospores and serves to extend their dispersal far beyond the mother colony. The slower mode of motility was observed for a period just prior to the anterior flagellum initiating settlement by adhering to the substratum. It appeared that the zoospores might be nearing the end of their dispersal phase and slowing the search in order to facilitate settlement, which was expedited by a relatively inactive flagellum now serving a new function. A lot of flagellate algae including other genera of pelagophytes revolve, spin, or flutter when moving, and this is not unusual. We are unaware of another example of a species that has two modes of complementary motility to secure settlement, but it would make sense that settling flagellates would all have a mechanism to slow down to the point where they recognize and then facilitate adhesion to a surface. For example, rafting zoospores of several different divisions of algae slow down to recognize and then claim a spot in a developing raft, often using the flagella to spin the cells and press them into the substratum where the force releases an adhesive at the apical end of the cells (e.g., for discussion, see Callow et al., 1997).

### Zoospore settlement and colony formation

The development of settled zoospores into new colonies can occur by two very different mechanisms that together further the chances of dispersal and colonization. First, zoospores simply settle as described above, adhere, and divide to produce colonies of various sizes. This is a simple sequence of vegetative reproduction seen in other pelagophyte species that have a zoospore stage: disperse, settle, adhere, and divide. If environmental conditions are good, this is a fast and easy way to establish new colonies and disperse to new habitats.

The second method of colony formation relied on the formation of a second benthic stage consisting of sporangia containing cells that constantly divided and pumped out more benthic cells directly, thus eliminating the alternate zoospore stage. This mechanism would greatly increase the chances of survival and success in a dynamic habitat (sand) that is normally constantly moving. The benthic sporangia provided a more constant response to favorable conditions, producing cells that continually released, adhered, and either formed new colonies right away or generated new benthic sporangia. If bad conditions prevail in tide pools, where water movement covers benthic sporangia with sand, established colonies could divide to produce motile zoospores that would migrate upward toward the light and settle, reestablishing the benthic stages.

### Benthic cells and sporangium formation in *Revolvomonas*

The spontaneous three‐dimensional rotation of benthic cells and the simultaneous formation of a sporangial wall is an interesting phenomenon and strongly suggests that the cell is involved both in the synthesis of the wall and providing the materials required for the process. After zoospore settlement and adhesion, cells immediately started the three‐dimensional rotation within a stationary thin wall generated by the cell. This wall was initially tight against the rotating cell, but as the rotations continued, the wall became elongated and started to thicken, the new sporangial wall becoming more pronounced as discussed in the Results section. The rotating cells always had a portion of their cell surface closely abutting the forming wall, possibly secreting materials used to synthesize the wall and/or helping that process in some unknown way.

Pelagophyte cells have been shown to have a novel secretory system with two types of Golgi and Golgi‐derived vesicles that differ in morphology and function, one that produces the PT and the second that produces the extracellular materials for the synthesis of the mucilage and cell walls that surround many pelagophytes outside the PT (Wetherbee et al., 2022). The latter Golgi‐derived vesicles deposit their contents extracellularly in a novel mechanism. Vesicles approached the PM but were never seen to fuse with the PM; rather, the PM responded by synthesizing a new section of PM to surround the vesicle. In this manner, vesicles were secreted extracellularly in their entirety. Now positioned between the PM and PT, the vesicle membrane disintegrated, releasing the contents that presumably moved through the pores of the PT to participate in wall and mucilage formation (Wetherbee et al., 2022). It seems reasonable to suggest that this secretory system is involved in the synthesis of the sporangium wall, as the rotating cell or cells always have contact over a large part of their surface with the sporangial wall. We can only speculate what drives the rotations that seem necessary for the formation of the sporangium wall, and we know of no similar mechanism where spontaneous three‐dimensional rotations generate wall secretion by a single, isolated cell. We would suggest participation by actin and myosin, but that study is well beyond the scope of this paper.

### Fine structure of the *Revolvomonas* PT

Our definition of the PT of pelagophytes is as follows: a multilayered cell covering that encases entire cells except where flagella emerge in flagellates and zoospores, possessing one or two electron dense, perforated layers interspersed with less dense fibrillar layers that all together form the PT. Two distinctive types of PT have been reported for the class, a four‐ or five‐layered PT observed thus far only in genera of the Pelagomonadales and a thinner, two‐layered PT, restricted to genera of the Sarcinochrysidales (summarized in Table S1), now including *Revolvomonas* and the recently described *Plocamiomonas psychrophila* (Daugbjerg et al., 2024). Within the Sarcinochrysidales, there are two types of bilayered PT. One is a thin layer #2 (12–15 nm) as observed in *Glomerochrysis* (fig. 8g in Wetherbee et al., 2021) and *Sargassococcus* (fig. 11a in Wetherbee et al., 2022) that is only marginally thicker than the PM and gives the PT the appearance of train tracks. A thicker layer #2 can be in the range of 25–35 nm, for example in *Chrysoreinhardia* and *Andersenia*, or much thicker in *Sungminbooa*, *Glomerochrysis*, *Gazia australica*, and *Aureoumbra* (35–80 nm) where layer #2 dominates the PT (summarized in Table S1). In the PT of *Revolvomonas*, layers #1 and #2 followed the same contour and were of the same thickness (40–50 nm), while layer #1 was fibrillar and lightly stained and was located between the PM and layer #2, which was electron dense and packed with micropores.

### Biogeography and range extensions

Information on the biodiversity and distribution of pelagophyte species is intermittent at best and is largely determined by whether a geographic area has been sampled and pelagophytes reported on. The presence of diverse populations of sand‐dwelling pelagophytes has only been reported within the past decade, mainly in Australia, where they are ubiquitous and often include species previously reported only from the northern hemisphere. For example, *Aureoumbra geitleri* has only been reported from the type locality in the Canary Islands, Spain. We have now found the same species to be common in Australia, first in Victoria (Wetherbee et al., 2021), then Heron Island (this paper), the coast of New South Wales, and the east coast of Tasmania (R. Wetherbee, unpublished data). This distribution suggests that *Au. geitleri* might be found in other locations between Spain and Australia if anybody had the resources to look. Alternatively, this species might have been transported by adhering to international shipping or in a ship's ballast water, as has been speculated for a number of invasive species in Australia, including both seaweeds and microalgae (for review, Hallegraeff, 2015; Hewitt et al., 2004).

The first report of a pelagophyte on the GBR described a bloom of *Chrysocystis fragilis* (Schaffelke et al., 2004), a species that is common on reefs of the pacific island of Guam from where the species was described (Lobban et al., 1995). We also cultured and sequenced *C. fragilis* from Heron Island and speculate that it might be common on the GBR and other Pacific reef systems. We previously reported on *Chrysoreinhardia giraudii* from Heron Island (Wetherbee et al., 2022) and have also observed this species along the east coast of Australia, including Tasmania. Common in the northern hemisphere, *Chrysoreinhardia giraudii* has been reported from France, the Mediterranean Sea, the Canary Islands, the Adriatic Sea, the Red Sea, and the Hawaiian Islands (Han et al., 2018; Hoffmann et al., 2000), with no clear connection with Australia.

*Sarcinochrysis marina* was recently collected from the type locality and cultured, with gene sequences published (Han et al., 2018). Our Heron Island strain had the same gene sequences and morphology as the type. We are not aware of another report of *Sa. marina* from Australia or the southern hemisphere, although it may turn out to be widespread now that the type species has been characterized and can be compared to other suspect species. Finally, we have cultured the planktonic *Pelagomonas calceolata* from Heron Island on several occasions. Originally described as oceanic (Andersen et al., 1993), it has been observed to be truly cosmopolitan in distribution, including in open oceans the world over. We have observed and occasionally cultured *P. calceolata* all along the east coast of Australia, from tropical north Queensland to temperate Tasmania, and west to West Australia, where this species frequently turns up in the water that accompanies our tide pool sand collections.

In total, including *Pelagomonas calceolata*, there are nine pelagophyte genera now identified from Heron Island, or just over 40% of the 22 known genera, with representative species for both the major lineages. We have also described an additional seven new species from Heron Island for a total of 13 species or 37% of the total of 35 known species. We are aware of a further two new genera from the Pelagomonadales now being processed, one from Heron Island and another from Cairns on the north Queensland coast adjacent to the GBR (R. Wetherbee, unpublished data). It is noteworthy that our results come from only three collecting trips to Heron Island located near the southern tip of the GBR, a very small sample size from a reef system that extends north for ~2000 km and includes 2900 individual reefs, yet strongly suggests that the Pelagophyceae is well adapted for a sand‐dwelling existence on tropical reefs and that additional taxa remain to be discovered on the GBR and elsewhere.

## AUTHOR CONTRIBUTIONS

**Richard Wetherbee:** Conceptualization (equal); data curation (equal); investigation (lead); visualization (lead); writing – original draft (lead); writing – review and editing (equal). **Allison van de Meene:** Investigation (supporting); methodology (supporting); writing – review and editing (supporting). **Riyad Hossen:** Investigation (supporting); writing – review and editing (supporting). **Robert A. Andersen:** Investigation (supporting); resources (supporting); writing – review and editing (supporting). **Heroen Verbruggen:** Conceptualization (equal); data curation (equal); formal analysis (lead); funding acquisition (lead); investigation (supporting); methodology (supporting); project administration (equal); visualization (equal); writing – original draft (supporting); writing – review and editing (supporting).

## FUNDING INFORMATION

Our work was funded by the Australian Biological Resources Study (activity 4‐G046WSD to HV) and the Fundação para a Ciência e a Tecnologia (CEECIND:2023.06155 to HV).

## Supporting information


**Figure S1.** Phylogenetic tree of the 18S rRNA gene analyzed in isolation.
**Figure S2.** Phylogenetic tree of the *psa*A gene analyzed in isolation.
**Figure S3.** Phylogenetic tree of the *psa*B gene analyzed in isolation.
**Figure S4.** Phylogenetic tree of the *psb*A gene analyzed in isolation.
**Figure S5.** Phylogenetic tree of the *psb*C gene analyzed in isolation.
**Figure S6.** Phylogenetic tree of the *rbc*L gene analyzed in isolation.

**Table S1.** Summary of information on the perforated theca of different pelagophyte genera.
