# Supplementary material for: The Great Barrier Reef, a center for Pelagophyceae (Heterokontophyta) diversity, including a new genus and seven new species
Source: J Phycol. 2025 May 28;61(3):678–98. doi: 10.1111/jpy.70030 (PMC12168098; doi:10.1111/jpy.70030)
Supplement: Supplementary file 1 — Figure S1. Phylogenetic tree of the 18S rRNA gene analyzed in isolation. Figure S2. Phylogenetic tree of the psaA gene analyzed in isolation. Figure S3. Phylogenetic tree of the psaB gene analyzed in isolation. Figure S4. Phylogenetic tree of the psbA gene analyzed in isolation. Figure S5. Phylogenetic tree of the psbC gene analyzed in isolation. Figure S6. Phylogenetic tree of the rbcL gene analyzed in isolation. [file JPY-61-678-s001.pdf]

18S

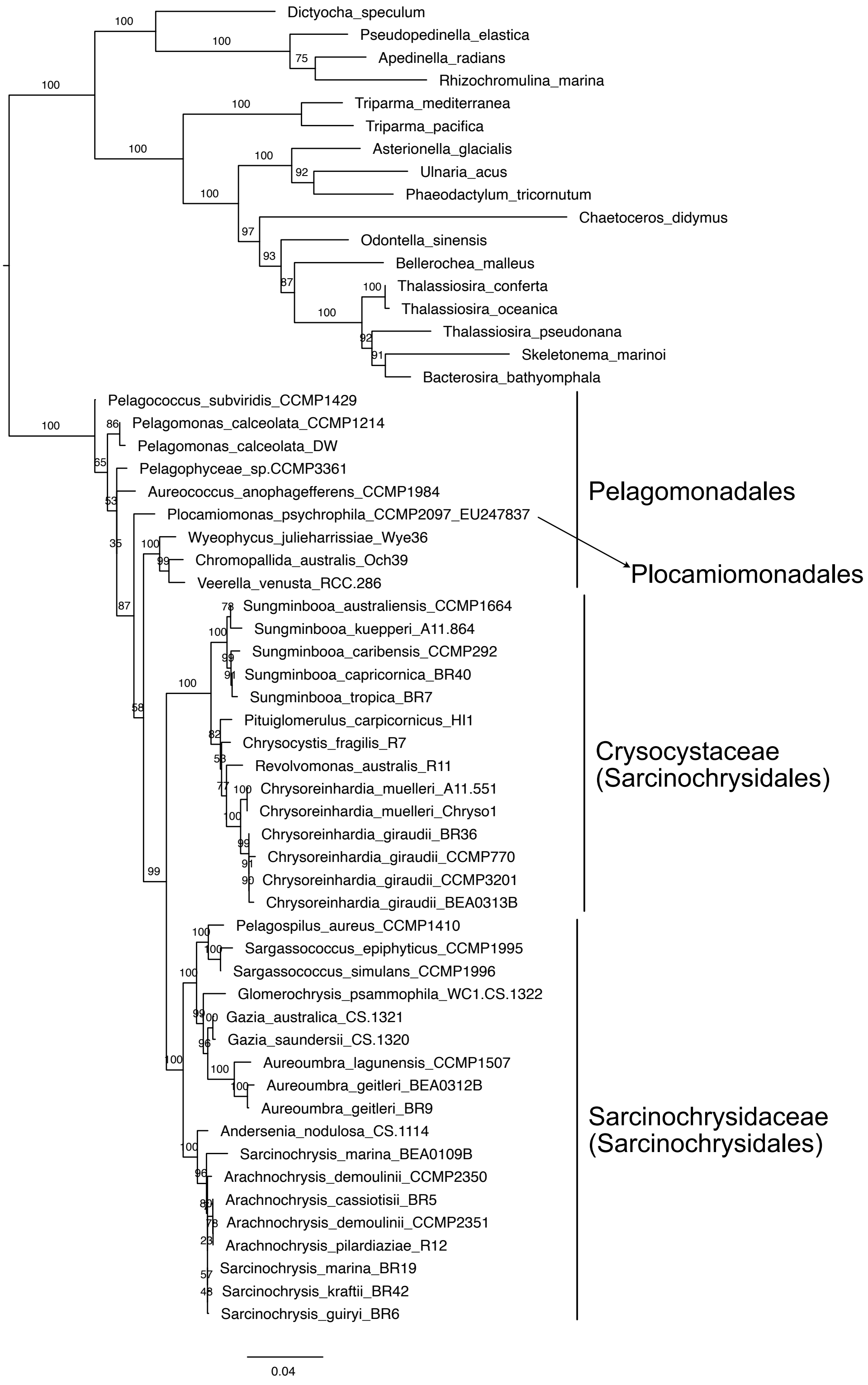

Figure S1. Phylogenetic tree of the 18S gene analysed in isolation.

*psa*A

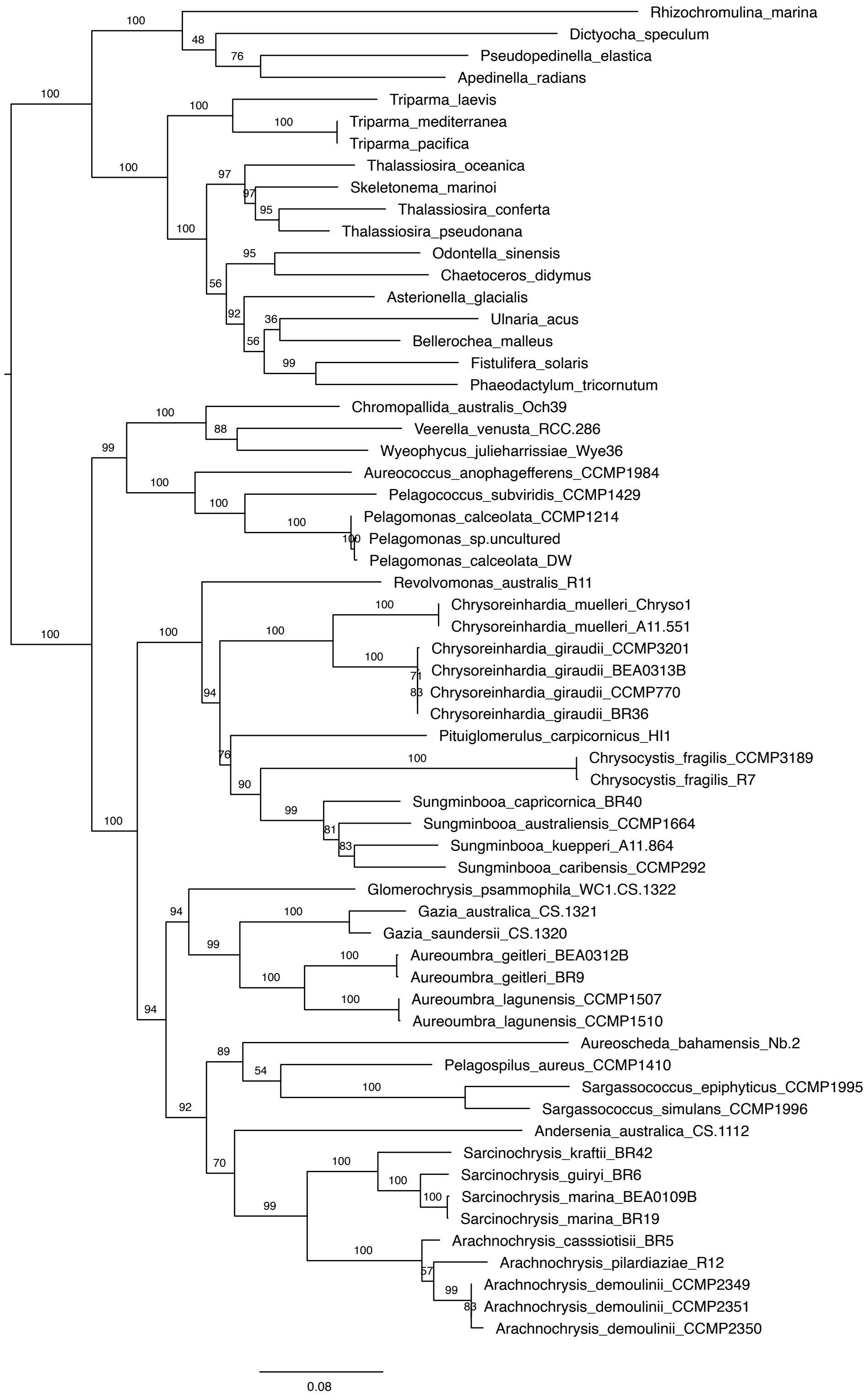

Figure S2. Phylogenetic tree of the *psa*A gene analysed in isolation.

*psa*B

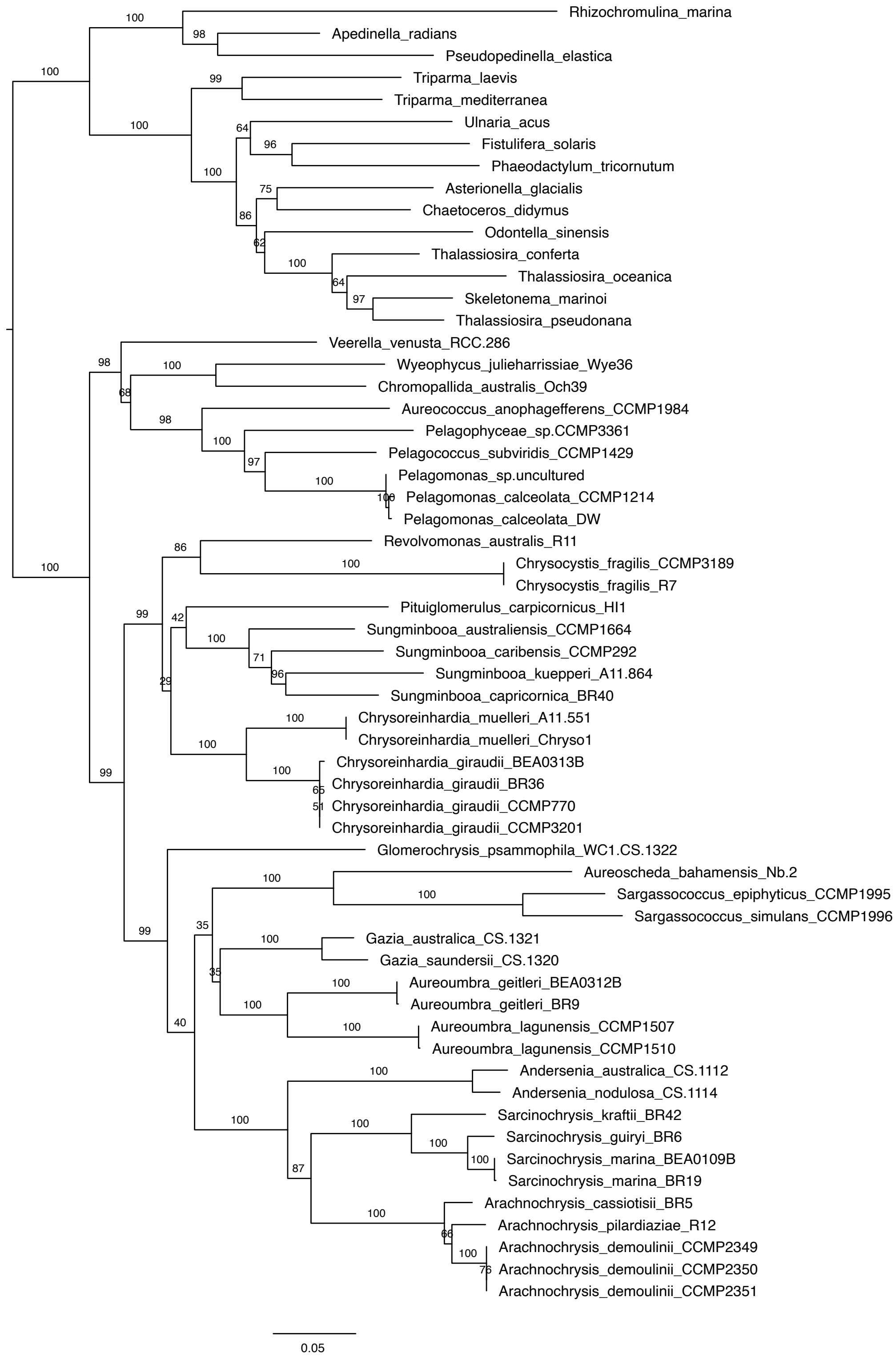

Figure S3. Phylogenetic tree of the *psa*B gene analysed in isolation.

*psb*A

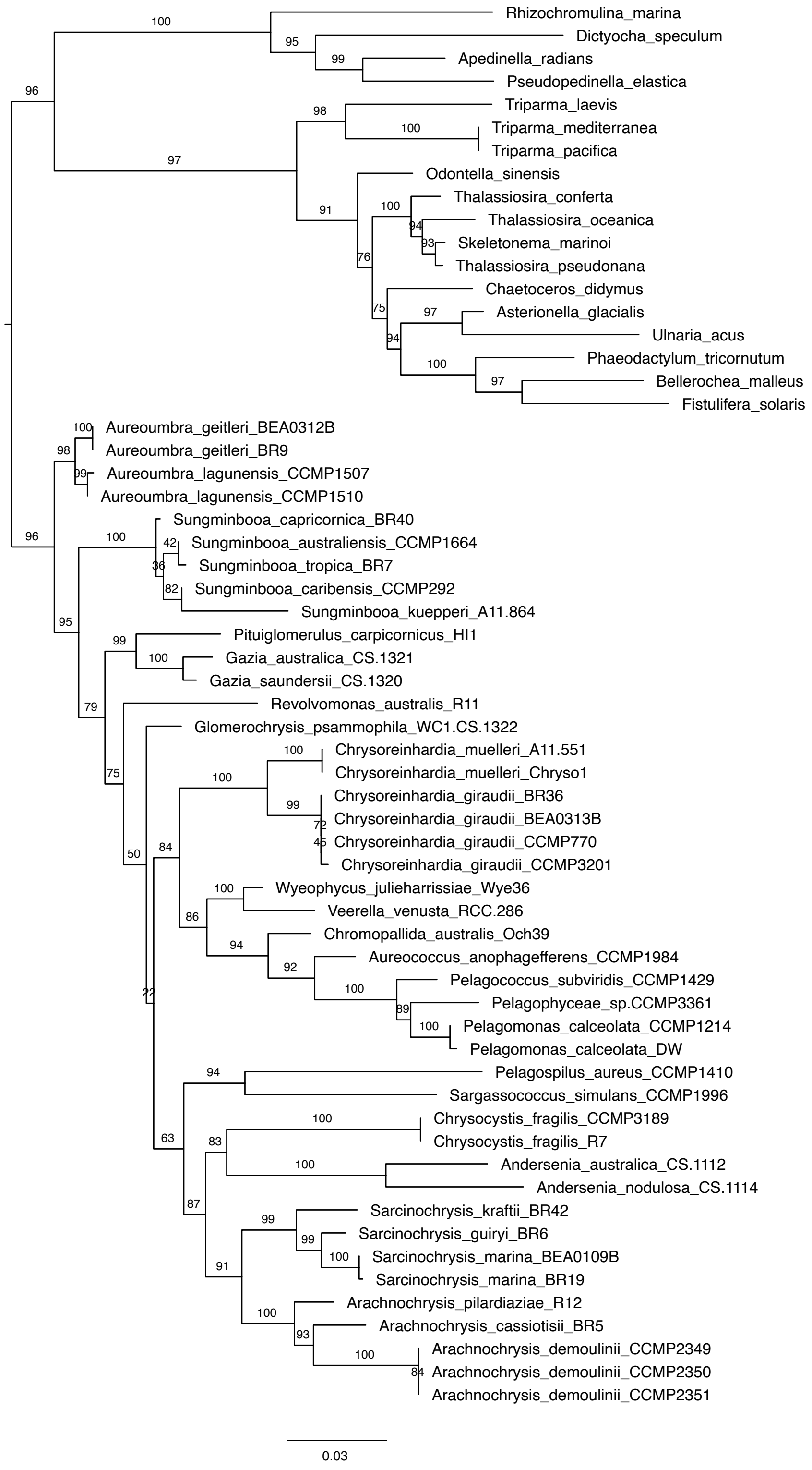

Figure S4. Phylogenetic tree of the *psb*A gene analysed in isolation.

*psb*C

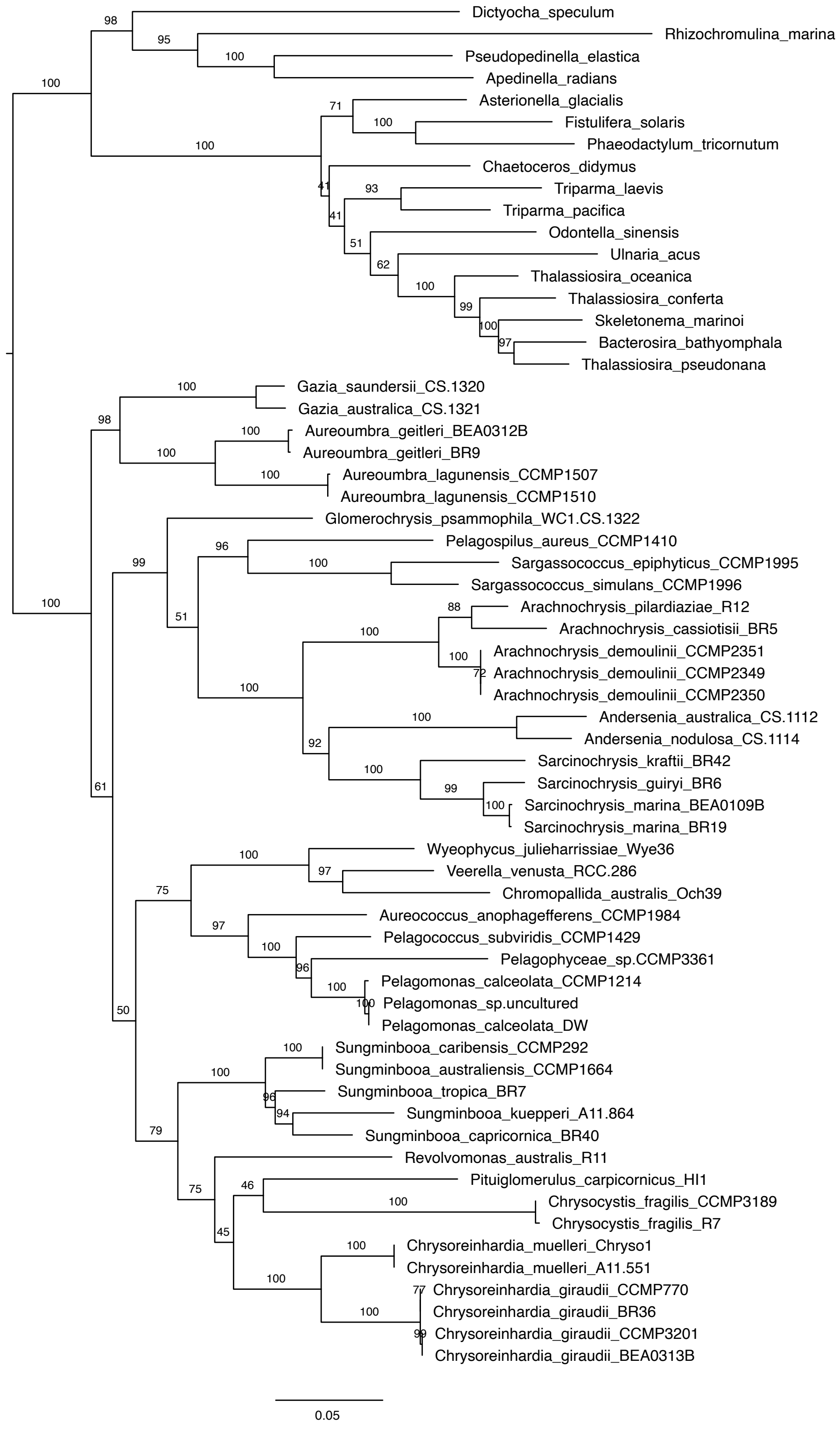

Figure S5. Phylogenetic tree of the *psb*C gene analysed in isolation.

*rbc*L

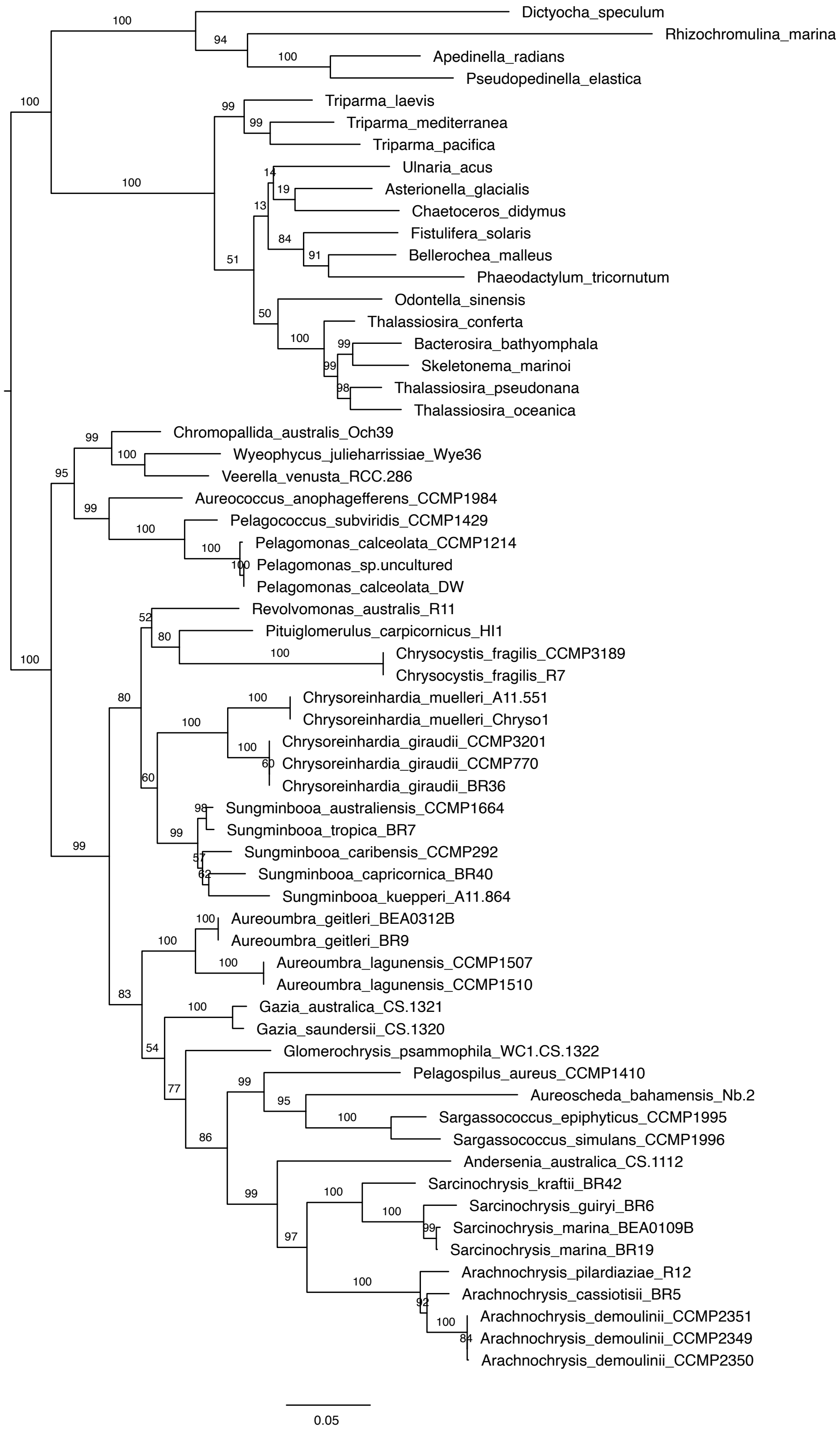

Figure S6. Phylogenetic tree of the *rbc*L gene analysed in isolation.
