# Supplementary material for: The Great Barrier Reef, a center for Pelagophyceae (Heterokontophyta) diversity, including a new genus and seven new species
Source: J Phycol. 2025 May 28;61(3):678–98. doi: 10.1111/jpy.70030 (PMC12168098; doi:10.1111/jpy.70030)
Supplement: Supplementary file 2 — Table S1. Summary of information on the perforated theca of different pelagophyte genera. [file JPY-61-678-s002.pdf]

Table S1. Perforated theca data (updated from Wetherbee et al. 2022). Data for the 14 pelagophyte genera studied thus far with TEM where information on the PT is available are listed, including the two species of *Gazia* where the PT is different. Note, the invalid name *Ankylochrysis* is replaced in this updated Table by *Veerella* (Andersen & Wetherbee 2024).

Taxon	PT Layers	PT Thickness	Layer #1	Layer #2	Layer #3	Layer #4	Layer #5	Pore types	Micropore size	Macropore size
**Pelagomonadales**										
*Wyeophycus**	5	120-140 nm	25-30 nm	20-30 nm	20-25 nm	20-30 nm	20 nm+	2	10--12 nm	20 -25 nm
*Chromopallida**	4	100-125 nm	20-25 nm	40-50 nm	20-25 nm	20-25 nm	none	1	10 -12 nm	18 -20. nm
Veerella	4	65-75 nm	c. 20 nm+	c. 25 nm	c. 20 nm	c. 12 nm	none	2	10 - 12 nm	18 - 20 nm
Pelagococcus	4	50-60 nm	20-25 nm	15-20 nm	15-20 nm	12-14 nm	none	1	12-14 nm	None
Aureococcus	4?	40-50 nm	?	?	?	?	none	?	?	?
Pelagomonas	1?	?	?	none	none	none	none	?	?	?
**Sarcinochrysidales**										
*Pituiglomerulus**	2	variable	variable	25-30 nm	none	none	none	1	none	20 -22 nm
*Chrysoreinhardia**	2	40-50 nm	12-18 nm	25-35 nm	none	none	none	1	10-12 nm	none
*Sungmumbooa**	2	45-55 nm	0-10 nm	45-55 nm	none	none	none	1	12-14 nm	none
*Glomerochrysis**	2	60-70 nm	20-24 nm	40-45 nm	none	none	none	1	none	18-22 nm
*Aureoumbra**	2	45-55 nm	12-15 nm	35-45 nm	none	none	none	1	none	18-20 nm
*Gazia saundersia**	2	25-30 nm	12-15 nm	12-15 nm	none	none	none	1	none	18-22 nm
*Gazia australica**	2	70-90 nm	10-12 nm	60-80 nm	none	none	none	2	5-8 nm	20-22 nm
*Sargassococcus**	2	30-40 nm	20-24 nm	12-14 nm	none	none	none	1	none	18-22 nm
*Andersenia**	2	45-55 nm	20-25 nm	25-30 nm	none	none	none	1	10-12 nm	none
*Revolvomonas****	2	80-100 nm	40-50 nm	40-50 nm	none	none	none	1	10-12 nm	none
*Plocamiomonas***	2	15-25 nm	7-12 nm	9-13 nm	none	none	none	?	?	?

* PT information from Wetherbee et al. (2021, 2022)
** PT information from Daugbjerg et al. (2024)
*** PT information presented in the present paper
